# Cryptochrome and quantum biology: unraveling the mysteries of plant magnetoreception

**DOI:** 10.3389/fpls.2023.1266357

**Published:** 2023-10-04

**Authors:** Thawatchai Thoradit, Kanjana Thongyoo, Khwanchai Kamoltheptawin, Lalin Tunprasert, Mohamed A. El-Esawi, Blanche Aguida, Nathalie Jourdan, Kittisak Buddhachat, Marootpong Pooam

**Affiliations:** ^1^ Department of Biology, Faculty of Science, Naresuan University, Phitsanulok, Thailand; ^2^ State Key Laboratory for Mechanical Behavior of Materials, School of Material Science and Engineering, Xi’an Jiaotong University, Xi’an, China; ^3^ Botany Department, Faculty of Science, Tanta University, Tanta, Egypt; ^4^ UMR CNRS 8256 Adaptation biologique et vieillissement (B2A), Institute of Biology Paris Seine, Sorbonne Université, Paris, France

**Keywords:** cryptochrome, radical-pair mechanism, level crossing mechanism, magnetitebased magr, magnetoreception, quantum biology

## Abstract

Magnetoreception, the remarkable ability of organisms to perceive and respond to Earth’s magnetic field, has captivated scientists for decades, particularly within the field of quantum biology. In the plant science, the exploration of the complicated interplay between quantum phenomena and classical biology in the context of plant magnetoreception has emerged as an attractive area of research. This comprehensive review investigates into three prominent theoretical models: the Radical Pair Mechanism (RPM), the Level Crossing Mechanism (LCM), and the Magnetite-based MagR theory in plants. While examining the advantages, limitations, and challenges associated with each model, this review places a particular weight on the RPM, highlighting its well-established role of cryptochromes and *in-vivo* experiments on light-independent plant magnetoreception. However, alternative mechanisms such as the LCM and the MagR theory are objectively presented as convincing perspectives that permit further investigation. To shed light on these theoretical frameworks, this review proposes experimental approaches including cutting-edge experimental techniques. By integrating these approaches, a comprehensive understanding of the complex mechanisms driving plant magnetoreception can be achieved, lending support to the fundamental principle in the RPM. In conclusion, this review provides a panoramic overview of plant magnetoreception, highlighting the exciting potential of quantum biology in unraveling the mysteries of magnetoreception. As researchers embark on this captivating scientific journey, the doors to deciphering the diverse mechanisms of magnetoreception in plants stand wide open, offering a profound exploration of nature’s adaptations to environmental cues.

## 1 General introduction

The potential physiological significance of plants’ geomagnetic response is a multifaceted and intriguing subject that has garnered attention in recent scientific explorations. The geomagnetic field, a natural environmental factor, exhibits considerable regional variations in strength and direction, and its influence on living organisms, including plants, has been a subject of study since the observations of Louis Pasteur in 1862 and Savostine in 1930.

One of the fascinating aspects of this field of study is the ability of plants to perceive and respond to varying magnetic fields. A study by Shine et al. Davies (2023) has shed light on this phenomenon, demonstrating that plants can alter their gene expression and phenotype in response to specific features of the geomagnetic field. This finding opens up new avenues for understanding how geomagnetic triggers could lead to phenotypic changes in plants.

The effects of magnetic fields on plant growth and development have been extensively reviewed, with a focus on the complex nature of magnetic fields’ action on plant growth. Silva and Dobránszki (2015) discussed the physiological and biochemical responses of plants to magnetic fields, ranging from nanoTesla to geomagnetic levels. Their review underscored the complexity of this subject and the need for a more robust mathematical framework to understand these effects. Pioneer studies on plant physiological responses to different magnetic fields have explored the effects of static magnetic fields, electromagnetic fields, and various magnet alloys on plants (Abhary and Akhkha, 2023). These studies have laid the groundwork for understanding the intricate relationship between magnetic fields and plant physiology.

The underlying physiological mechanisms of magnetic fields’ effects on plants have also been probed. For instance, Xu et al. (2016) and Agliassa et al. (2018a) discovered that the flowering of *Arabidopsis* was suppressed by a near-null magnetic field, a phenomenon related to the modification of cryptochrome. This study also revealed significant decreases in gibberellin levels in plants grown in a near-null magnetic field compared to those in the local geomagnetic field, highlighting the nuanced ways in which magnetic fields can influence plant development. The metabolic processes of plants, including the vital process of photosynthesis, can be altered by geomagnetic disturbance. Davies (2023) observed a decrease in diurnal oxygen production in Elodea plants under high geomagnetic variability, suggesting that geomagnetic disturbance can affect the metabolic process of photosynthesis. Furthermore, the influence of the geomagnetic field on the metabolism of living organisms, including plants, has been supported by a plethora of studies (Erdmann et al., 2017). These investigations have revealed that any weakening or absence of the geomagnetic field can interfere with metabolic processes, adding another layer of complexity to our understanding of geomagnetic effects on plants. Electrical signals in plants, such as action potentials, have also been implicated in physiological processes (Fromm and Lautner, 2006). The presence of these electrical signals in plant cells indicates that plants use ion channels to transmit information over long distances, triggering rapid responses to environmental stimuli and affecting various physiological processes. The physiological significance of plants’ geomagnetic response extends to seed germination and growth as well. Studies by Abhary and Akhkha (2023) have found that magnetic fields influence seed vigor, growth, and yield, suggesting that magnetic fields could be harnessed to enhance plant growth and maximize crop performance.

Overall, the potential physiological significance of plants’ geomagnetic response is a rich and complex subject that encompasses various aspects of plant growth, development, and physiological processes. The existing body of research has provided valuable insights into how plants perceive and respond to magnetic fields and how these fields influence physiological and biochemical processes. However, the intricate nature of these effects and their significance in plant biology calls for further research to unravel the underlying mechanisms and to explore new frontiers in this fascinating area of study.

In the developing of plant science, the search to comprehend plants’ response to the Earth’s magnetic field offers an intriguing and multi-disciplinary exploration at the interface of quantum biology and molecular botany. Despite substantial progress in this domain, plant magnetoreception remains covered in scientific doubt, captivating researchers worldwide. The essence of this fascinating mystery lies within the underlying mechanisms that activate such responses, with the radical pair mechanism (RPM) emerging as a dominant candidate. This intriguing phenomenon controls the principles of quantum physics, blurring the lines between these seemingly distinct fields of science. The RPM theory posits that specific light-activated molecules, such as the flavoprotein cryptochrome, create pairs of free radicals whose spin states and hence chemical reactivity may be influenced by Earth’s magnetic field (Ritz et al., 2000). This intriguing hypothesis has profound implications for plant biology, suggesting that the cryptochrome-mediated light sensing mechanism in plants could serve as a biological compass, shaping critical processes such as growth, germination, and phototropism (Ahmad, 2016). Moreover, quantum biology’s contribution to our understanding of magnetoreception might be further enhanced through the Level Crossing Mechanism (LCM), which proposes that magnetic fields can influence energy level transitions in molecules or atoms, potentially contributing to magnetoreception in plants. However, despite being a novel concept, the LCM has received limited attention compared to RPM, and its potential role in plant magnetoreception remains largely unexplored.

Delving deeper into this enigmatic phenomenon, the magnetoreceptor protein MagR emerges as another promising player (Qin et al., 2016). A recent study by (Parmagnani et al., 2022) postulates a potential role for MagR in plant magnetoreception, with the protein possibly forming complexes with iron-sulfur (Fe-S) clusters in response to geomagnetic fields. This line of thought represents a novel nexus between RPM and MagR theories, introducing the idea of a composite magnetoreceptor that integrates elements of light sensitivity and magnetic field alignment (Parmagnani et al., 2022). However, the MagR theory is not without its challenges. Empirical support is limited, and the relationship between MagR and cryptochrome, as well as their role within the RPM or magnetite-based mechanism, remains elusive. Notwithstanding these challenges, unraveling the role of MagR could provide an indispensable bridge between the domains of quantum biology and plant magnetoreception (Dodson et al., 2013).

As our understanding of plant magnetoreception continues to unfold, it becomes increasingly evident that we must inspect the complicated involvement between quantum physics and biology. From cryptochrome to MagR, the potential players are numerous, and their intricate interplay paints a complex picture of plant magnetoreception.

However, each mechanism that we mentioned above is still under debate. Thus, the objective of this review is to revisit the current understanding of plant magnetoreception, exploring both well-established and emergent theories, with a particular focus on the RPM and the potential role of quantum biology. By illuminating the potential intersections between these theories and suggesting avenues for future research, we aim to advance our collective understanding of this extraordinary phenomenon. As we embark on this journey, let us marvel at the wonders of nature, where the smallest seed can grasp the vast forces of our planet, driven by the unseen, yet undeniable influence of Earth’s magnetic field.

## 2 Quantum biology and magnetoreception

Quantum biology, an emerging interdisciplinary science, integrates the principles of quantum mechanics into understanding biological phenomena. This integration may initially seem paradoxical, but it possesses the potential to illuminate many complicated biological processes that remain unexplained by classical biology (McFadden and Al-Khalili, 2016). Quantum mechanics, the heart of quantum biology, explores into the science of nanoscopic particles at the atomic and subatomic levels. Governed by rules that often appear counterintuitive in everyday life, these particles form the foundation of quantum theory and its potential applications to biological phenomena (Berman, 2016).

One such application pertains to the understanding of magnetoreception, an elusive sensory mechanism enabling organisms to detect and respond to Earth’s magnetic fields. Quantum processes may play a critical role in this phenomenon (Wiltschko and Wiltschko, 2019). The Key aspects of quantum mechanics integral to understanding quantum biology are the concept of *quantum entanglement and quantum coherence*. The concept of *quantum entanglement* denotes a phenomenon where the quantum state of each particle in a pair or group cannot be described independently of the others, regardless of the distance separating them. Providing an accessible metaphor for one of the most confusing yet fundamental concepts in quantum entanglement, imagine two twins who are far apart, one in Bangkok and the other in Paris. If something happens to one twin, the other instantly knows, even though they are thousands of kilometers apart. This illustrates quantum entanglement, where the state of one particle instantly influences the state of another, regardless of their spatial separation. This phenomenon has been confirmed experimentally (Castelvecchi and Gibney, 2022).

In another player, *quantum coherence*, describes the ability of a quantum system to maintain this superposition state over time (Schlosshauer, 2007). To create a clear picture, imagine a coin spinning in mid-air. While it’s spinning, you could argue that it could be both heads and tails at the same time, which is similar to a superposition. One might compare quantum coherence to the theoretical scenario in which a spinning coin, suspended mid-air, maintains its rotation indefinitely without committing to a particular orientation, be it heads or tails.

The principle of quantum entanglement and quantum coherence applies to the RPM as a theory in quantum biology. For more detail, RPM involves a photochemical reaction where light excitation creates a pair of radicals, or molecules with an unpaired electron. These radicals are quantum entangled, and their properties show that their spins can mirror each other. The introduction of a magnetic field causes these spins to flip at different rates, disrupting their synchrony. An organism can detect this disturbance, thereby sensing the magnetic field. Thus, RPM is suggested as a candidate mechanism for magnetoreception in living organisms, and cryptochrome proteins are hypothesized to mediate this process (Ritz et al., 2000).

Cryptochromes, proteins that regulate light-dependent physiological processes, are believed to contribute to magnetoreception in various organisms, including plants (Hammad et al., 2020). When exposed to blue light, quantum effects within cryptochromes might trigger a series of biochemical reactions that could potentially influence plant responses to magnetic fields. However, our understanding of plant magnetoreception remains in its beginning, with most experimental evidence derived from studies on *Arabidopsis thaliana* (Pooam et al., 2020). Exposure to magnetic fields elicited responses in these plants, possibly mediated by cryptochromes. Nonetheless, the precise mechanism remains speculative, underscoring the need for further rigorous research. Additionally, not all plant responses to magnetic fields can be explained by the RPM. Another possible quantum-based mechanism in plant magnetoreception has also been reported. The research suggests that *Arabidopsis thaliana* has mechanisms for detecting magnetic fields that do not rely on light and may be more consistent with LCM of magnetoreception than the cryptochrome-associated RPM (Dhiman et al., 2023). LCM is a concept in quantum mechanics that explains how certain systems undergo transitions between different energy levels. It is particularly relevant in the study of magnetoreception. This suggests that multiple pathways may exist for magnetoreception in plants, highlighting the complexity of these processes.

Synthesizing the above, the fusion of quantum mechanics and biology expands our understanding of natural phenomena in unanticipated ways. The intricate dance of quantum particles may be orchestrating more biological processes than we currently comprehend. As we continue to uncover these hidden connections, we could revolutionize our understanding of life’s fundamental processes.

## 3 The quantum-based magnetoreception mechanism

According to the quantum biology concepts we mentioned above, the study of magnetoreception, has spurred a plethora of exciting theories. Significantly, two significant quantum-based theories are proposed, the RPM and the LCM. The subsequent sections will provide an easily understandable introduction to these mechanisms.

### 3.1 Radical-pair mechanism: illuminating nature’s quantum compass

**Figure 1:**
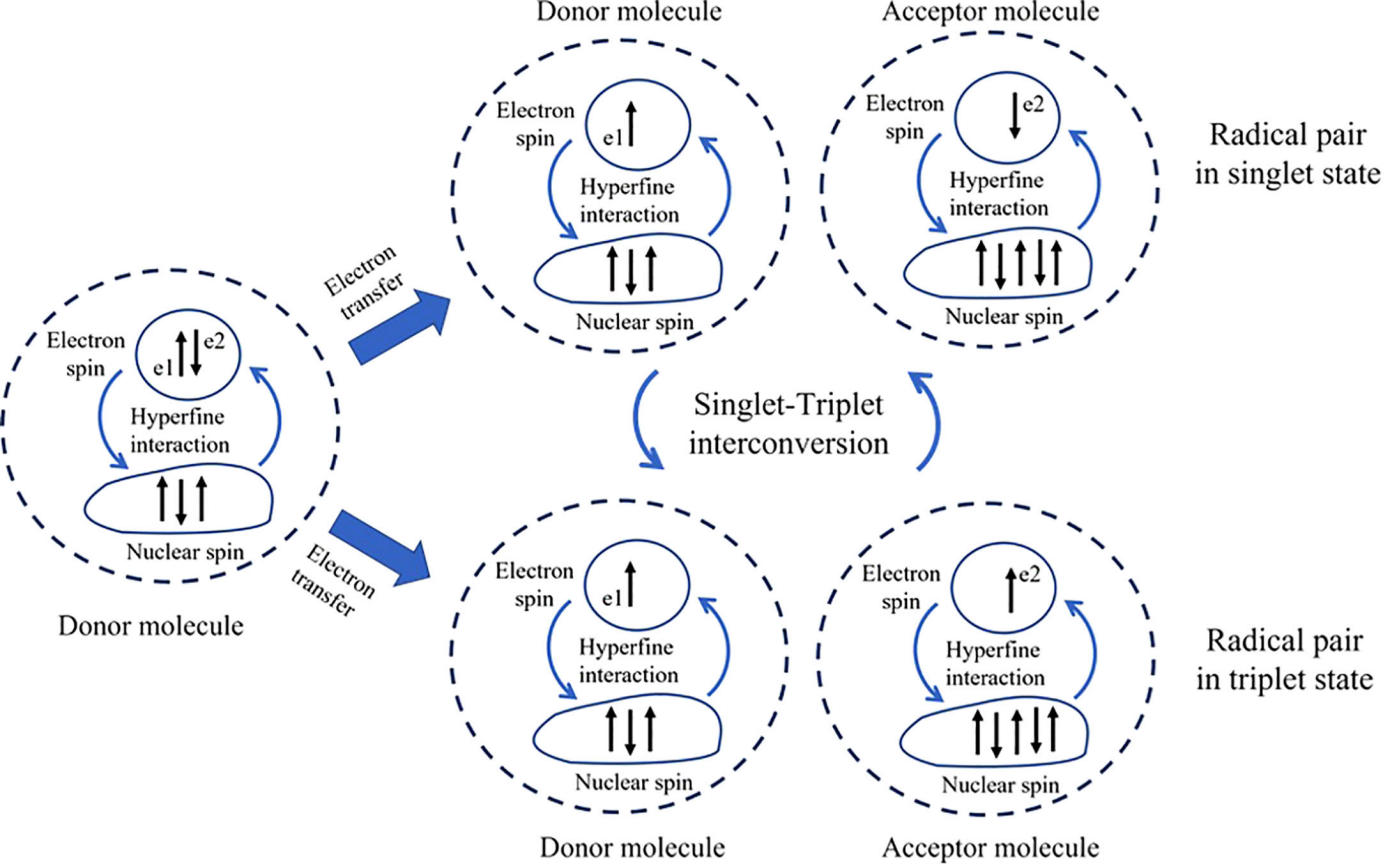
Nuclear-spin and the singlet-triplet interconversion. The donor molecule, which has three nuclear spins, transfers one electron (e2) to the acceptor molecule, which has four nuclear spins. The difference in hyperfine interaction resulting from the number of nuclear spins can induce the electron’s spin to be in a single or triplet state and facilitate interconversion between them. The dashed line between e1 and e2 represents the correlation between the two electrons.

The RPM, an early quantum-based theory proposed over 40 years ago (Schulten et al., 1978), represents a fascinating intersection where quantum physics and biology meet. This intersection opens new possibilities in the field of quantum biology, aiming to crack the mysteries of magnetoreception. The RPM, suggested by Schulten and his team in 1978, explains how Earth’s low-energy magnetic fields can interact with biochemical reactions in organisms (Schulten et al., 1978) even though the energy of magnetic fields is lower than thermal energy. This mechanism involves two radical molecules, electron donor and electron acceptor molecule (**Figure 1**), each produced from a chemical reaction within the organism. Radicals are characterized by an odd number of electrons, contributing to their instability. A defining feature of these radicals is the spin magnetic moment of their unpaired electron. The essential element in this mechanism is the quantum mechanical entanglement of the spins of the unpaired electrons in these two radicals. Intrinsically linked, the state of one instantaneously influences the state of the other, regardless of the distance between them. This entanglement gives rise to the phenomenon of quantum coherence in the radical pair system. Each radical molecule’s spin direction can randomly orient itself in either a parallel (triplet) or anti-parallel (singlet) state. The transition between these states, known as singlet-triplet interconversion, is facilitated by hyperfine interaction—an interaction between the nuclear spin (resulting from the spinning motion of protons and neutrons) and the spin of the unpaired electron (Hore and Mouritsen, 2016). This superposition state of singlet and triplet state could refer as the superposition state and it can oscillate between these two states due to the intrinsic properties of quantum mechanics as called, quantum coherent spin dynamics. The rate of conversion between singlet and triplet states tends to remain stable at a specific frequency pattern. Interestingly, the introduction of an external magnetic field interacts with the spin magnetic moments of these radicals, causing them to flip or alter their spins at different rates. This interaction with the unpaired electron, known as the Zeeman interaction, can change the dynamics of the interconversion, affecting the rate and final yield of the reaction. This disruption in their synchrony can be detected by the organism, thus forming the basis for magnetoreception (Hore and Mouritsen, 2016). In summary, the quantum coherence in the RPM is crucial because it allows the radical pair to exist in a superposition of spin states. This superposition enables the radical pair to be sensitive to the surrounding magnetic field. The external magnetic field can influence the relative spin alignment of the radical pair, leading to different reaction outcomes depending on the orientation and strength of the magnetic field. This interference is hypothesized to be sensitive to very weak magnetic fields, such as the Earth’s magnetic fields (Lambert et al., 2012). For further detail, we recommend reading one of the most excellent papers in this field: Hore and Mouritsen (2016).

However, this mechanism is limited by spin relaxation. Over time, these spins undergo relaxation, lose this orientation, and return to a random, equilibrium state. The spin-relaxation time of each radical pair, determined by their hyperfine interactions, varies among different radical pairs. This factor is crucial when considering the potential magneto-sensitivity of a biological molecule. Moreover, another key challenges in quantum biology, and particularly in the RPM, is the issue of maintaining quantum coherence and entanglement in a warm and noisy biological environment. These quantum states are delicate and can be easily disrupted—a process known as decoherence. Overcoming this issue of decoherence to allow for meaningful biological effects is one of the significant hurdles to be crossed in the field of (Schlosshauer, 2007).

The study of quantum effects in biological systems like the RPM in magnetoreception is a relatively new field. However, the insights it offers could revolutionize our understanding of biological processes and necessitate a paradigm shift in the way we approach biological research.

Indeed, magnetoreception in migratory animals and the underlying radical-pair mechanism have been recognized since the 1970s, but the primary magnetosensitive molecule remains elusive. In the next section, we will discuss cryptochrome, the candidate magnetoreceptor from the radical-pair mechanism.

### 3.2 Cryptochromes: the enigmatic puzzle of magnetoreception

Scientists, in their quest to identify a viable biological molecule for magnetoreception, imagine a molecule within a living organism that can absorb external energy and create a suitable radical pair. This pair should be capable of interacting with external magnetic fields. Light, as one of the most frequent external energies, can induce a biological molecule to produce a radical pair in the living organism. Consequently, this phenomenon leads to the conjecture that photoreceptors could function as magnetosensing sites A significant breakthrough was achieved in 1992 when researchers discovered that migratory birds orient themselves towards the magnetic direction under blue or green light, but not red light. This implies that the photopigment for magnetoreception should be sensitive to these colors (Wiltschko et al., 1993). In more detail, the avian magnetic compass is influenced by light, functioning effectively in bright sunlight and under full-spectrum light (Wiltschko and Wiltschko, 1981). It has been found that short-wavelength light is essential, and the required light intensities are surprisingly low. Orientation experiments with birds have shown sensitivity to wavelengths such as 373 nm (UV), 424 nm (blue), 501 nm (turquoise), and 565 nm (green). However, wavelengths such as 568 nm and 585 nm (yellow) and 617 nm, 635 nm, and 645 nm (red) led to disorientation (Wiltschko et al., 1993; Wiltschko and Wiltschko, 1995; Wiltschko and Wiltschko, 1999; Pinzon-Rodriguez and Muheim, 2017). The sensitivity of migratory birds to specific wavelengths, particularly in the blue and green regions of the spectrum, underscores the intricate relationship between light and navigation. This phenomenon may be linked to the birds’ ability to perceive the Earth’s magnetic field, with blue and green light potentially playing a role in enhancing this perception. For navigation, birds may need to have photoreceptors that can perceive short-wavelength light such as 373 nm (UV), 424 nm (blue), and 501 nm (turquoise), allowing them to respond to these specific wavelengths. The observed disorientation under yellow and red light suggests a finely tuned mechanism that relies on specific wavelengths for orientation.

Concurrently, the blue-light photoreceptor cryptochrome was discovered in plants (Ahmad and Cashmore, 1993). Cryptochrome was subsequently incorporated into the radical-pair mechanism by Schulten and colleagues, which was in line with their theory. In 2000, Schulten and Ritz proposed that cryptochrome is a potentially magnetically sensitive molecule in the radical-pair mechanism, implying a crucial role for eyes and light in the magnetic compass (Ritz et al., 2000).

For more information, cryptochromes are flavoproteins fundamental to a multitude of biological processes in various organisms, notably in *Arabidopsis* thaliana, where they were first discovered (Ahmad and Cashmore, 1993). They regulate numerous functions in plants, such as circadian rhythms, photoperiodism, flowering, and hormone signaling (Chaves et al., 2011). These proteins bear a structural resemblance to photolyases, enzymes involved in DNA repair, thereby suggesting a common ancestral origin (Chaves et al., 2011). Chaves et al. (2011) reviewed the primary structure of cryptochromes which consists of an N-terminal photolyase-related region and a divergent C-terminal domain. The photolyase-related region encompasses the chromophore binding site: the flavin adenine dinucleotide (FAD). It is within this region that the crucial radical pair interactions central to the proposed magnetoreception mechanism occur. The FAD molecule is thought to be the site where the radical pairs form in the magnetoreception process. Following the absorption of a photon, an electron from the FAD molecule is excited into a higher energy state, resulting in the formation of a radical pair involving FAD and a nearby tryptophan (Trp) residue (Hore and Mouritsen, 2016). These radical pairs, entangled and subsequently existing in a quantum superposition of singlet and triplet states, can be influenced by Earth’s magnetic field. This influence, in turn, affects the interconversion between these states and the overall yield of reaction products. The C-terminal domain of cryptochromes, which is essential for signal transduction, contains a series of protein-protein interaction motifs that enable cryptochromes to participate in various signaling pathways. Thus, once the magnetic information is perceived and processed via the RPM, the C-terminal domain aids in translating it into a physiological response (Ahmad, 2016). The structural complexity of cryptochromes, with their chromophore binding site and interaction motifs, emphasizes their versatility in sensing and responding to environmental cues, including magnetic fields. The unique capability of cryptochromes to form radical pairs, thereby facilitating magnetoreception, underscores their importance in the domain of quantum biology.

**Figure 2:**
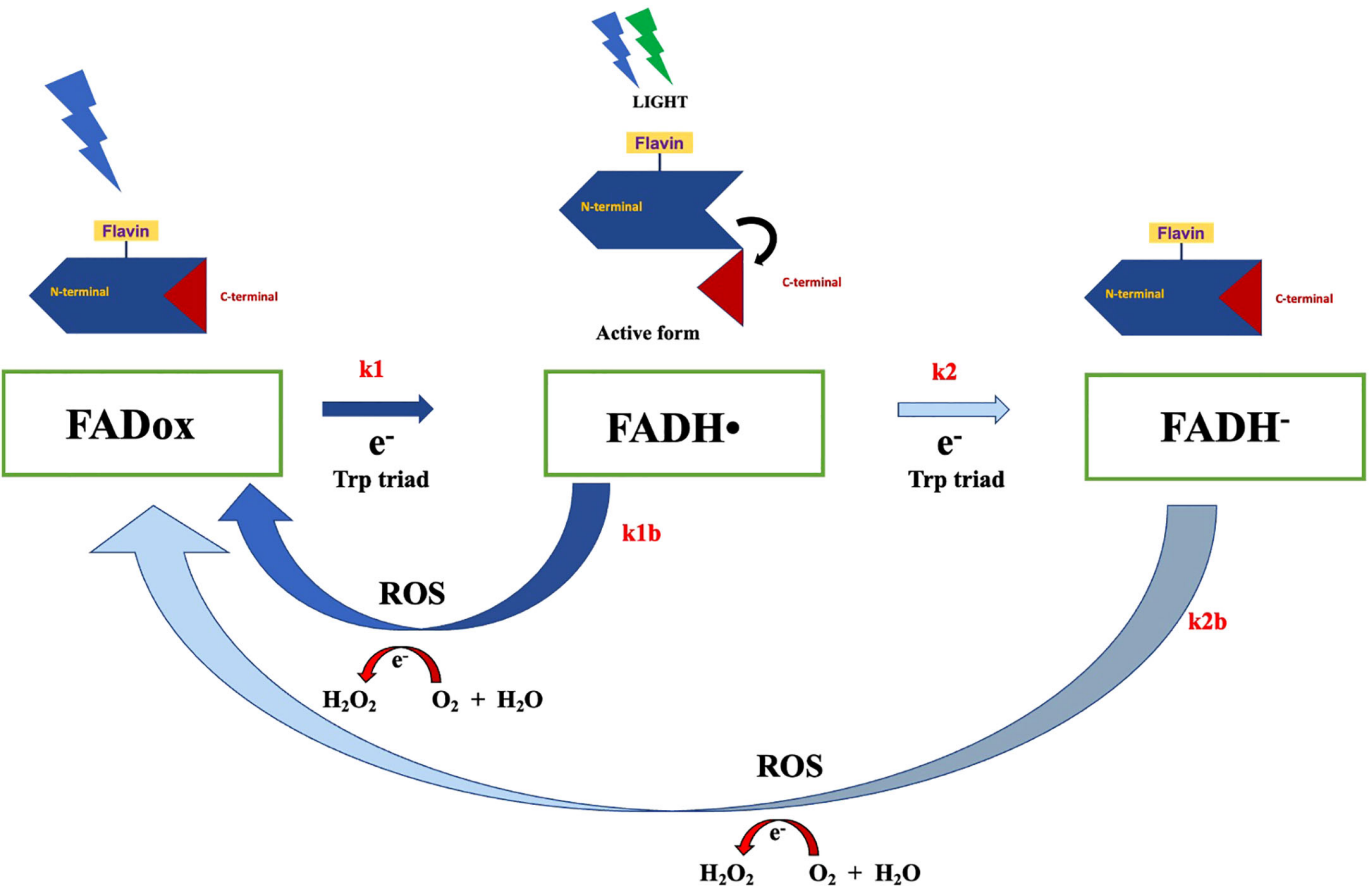
The *Arabidopsis* cryptochrome photocycle. In the dark, cryptochrome remains in an inactive state, with flavin in its oxidized form. Illumination with blue light can trigger flavin photoreduction, whereby FADox is reduced to FADH• (rate constant k1) through a forward electron transfer via the Trp triad pathway. This process induces conformational changes and the unfolding of the c-terminal domain, the signaling state of cryptochrome, allowing the protein to interact with signaling partners. Subsequent illumination of FADH• with blue or green light can prompt further photoreduction, wherein flavin in FADH• is reduced to the fully reduced state FADH-, at a constant rate k2. However, the reduced state of flavin can revert back to FADox via a reoxidation reaction, generating reactive oxygen species (ROS) and H_2_O_2_.

The activation of cryptochromes (**Figure 2**), particularly the photoreduction of FAD via the Trp triad pathway, induces a conformational change that allows cryptochrome to interact with protein partners for signal transduction response (Ahmad, 2016). This process also leads to alterations in the C-terminal extension, facilitating interaction with signaling partners such as CIB1 (Liu et al., 2017). As these interactions can result in protein phosphorylation during the activation state, they can significantly influence plant development. Interestingly, cryptochrome reoxidation generates reactive oxygen species (ROS), which could potentially contribute to magnetoreception response (Ahmad, 2016). Cryptochromes have also been proposed as potential magnetoreceptors in animals (Fedele et al., 2014a; Fedele et al., 2014b; Wiltschko et al., 2021), particularly in avian navigation (Ritz et al., 2000; Wiltschko and Wiltschko, 2019). Experimental evidence aligns cryptochromes with the radical-pair mechanism, positioning them as integral to magnetoreception (Gegear et al., 2008; Foley et al., 2011; Bazalova et al., 2016). Despite the compelling evidence supporting the role of cryptochromes in animal magnetoreception, their function in plant magnetoreception is not yet thoroughly established. Experimental results in this context are insufficient to provided comprehensive insight into plant magnetoreception mechanisms mediated by cryptochromes. Further studies are, therefore, warranted to elucidate this understudied aspect of plant physiology.

### 3.3 Exploring the potential of cryptochrome as a key player in plant magnetoreception

Magnetoreception in plants, although a relatively underexplored domain, has sparked considerable interest due to its complex interplay with the Earth’s magnetic fields. Certain effects on plant development, as a result of magnetic field exposure, have been documented (Maffei, 2014). For example, pea epicotyls were found to be longer under low magnetic field conditions, potentially due to increased cell elongation and osmotic pressure (Yamashita et al., 2004). These findings suggest that magnetoreception plays a role in plant growth and development, although the specific mechanisms require further investigation. The influence of magnetic fields, both weak and strong, on plant physiology opens exciting new avenues for research in plant sciences (Maffei, 2014).

Regarding the mechanism, magnetic fields affect cryptochrome-dependent responses, including hypocotyl growth and anthocyanin accumulation, as well as influencing on the expression of related genes (Xu et al., 2012; Xu et al., 2014; Xu et al., 2017; Xu et al., 2018; Pooam et al., 2019). Unquestionably, experiments conducted in some studies also demonstrate the direct effect of magnetic fields on the phosphorylation on the C-terminal domain of cryptochromes (Pooam et al., 2019; Albaqami et al., 2020; Hammad et al., 2020). To evaluate the response of C-terminal phosphorylation to the magnetic field, we conducted phosphorylation experiments utilizing Western blotting techniques. Cryptochrome phosphorylation was visualized on the Western blots, evident through an upward mobility shift of the phosphorylated protein. We analyzed the intensity of the phosphorylated band using ImageJ software, following the methodologies described by Pooam et al. (2019) and Albaqami et al. (2020). Our findings revealed that the phosphorylation of cryptochrome was modulated in response to the applied magnetic field (Pooam et al., 2019; Albaqami et al., 2020). This increase in phosphorylation serves as a direct indication of enhanced cryptochrome biological response and activation and substantiates the role of cryptochrome in magnetoreception.

Nonetheless, other photoreceptors, such as phytochromes, might also be influenced by geomagnetic fields (Agliassa et al., 2018b; Dhiman and Galland, 2018). While the evidence points towards a significant role of cryptochromes in magnetoreception, their exact contribution, whether as primary magnetoreceptors or functioning in tandem with other molecules, remains to be definitively established. Future research is essential to fully elucidate this complex process.

To find a resolution to this debate, another approach to provide evidence that cryptochromes function as magnetoreceptors involves the use of radiofrequency (RF) as a diagnostic tool. The application of RF fields to investigate the role of cryptochromes in magnetoreception is based on their capacity to interfere with the dynamics of radical pairs. Specifically, radiofrequency fields can induce additional flips in the spins of the electrons within the radical pairs, thereby disrupting the magnetic information (Ritz et al., 2009). Within the framework of the RPM, the alignment of electron spins in the radical pair is susceptible to external magnetic fields. These fields can induce the flipping of spins, which modifies the interconversion rate between singlet and triplet states. The organism can detect these changes, which form the basis for magnetoreception. The introduction of an RF with a suitable frequency - known as the Larmor frequency, which is determined by the gyromagnetic ratio of the electron and the local magnetic field strength - can trigger additional spin flips, essentially scrambling the magnetic information. This occurs because RF, like the Earth’s magnetic field, contain a magnetic component that can interact with the electron spins (Engels et al., 2014). If the application of the RF impairs an organism’s ability to sense magnetic fields, it suggests that magnetoreception is likely mediated by a RPM. Experiments using radiofrequency fields have been performed on migratory birds, and the results strongly suggest that the birds’ magnetic compass operates based on a RPM, potentially involving cryptochromes (Ritz et al., 2009; Engels et al., 2014).

Moreover, findings from the study demonstrate a significant decline (up to 24%) in response to blue light in *Arabidopsis* seedlings exposed to radiofrequency (RF) fields. This provides compelling evidence to support the notion that cryptochromes are likely candidates to act as plant magnetoreceptors, within the context of the radical-pair mechanism (Albaqami et al., 2020).

### 3.4 Diving into the depths: decoding the dance of the radical-pair mechanism and photocycle in cryptochromes

**Figure 3:**
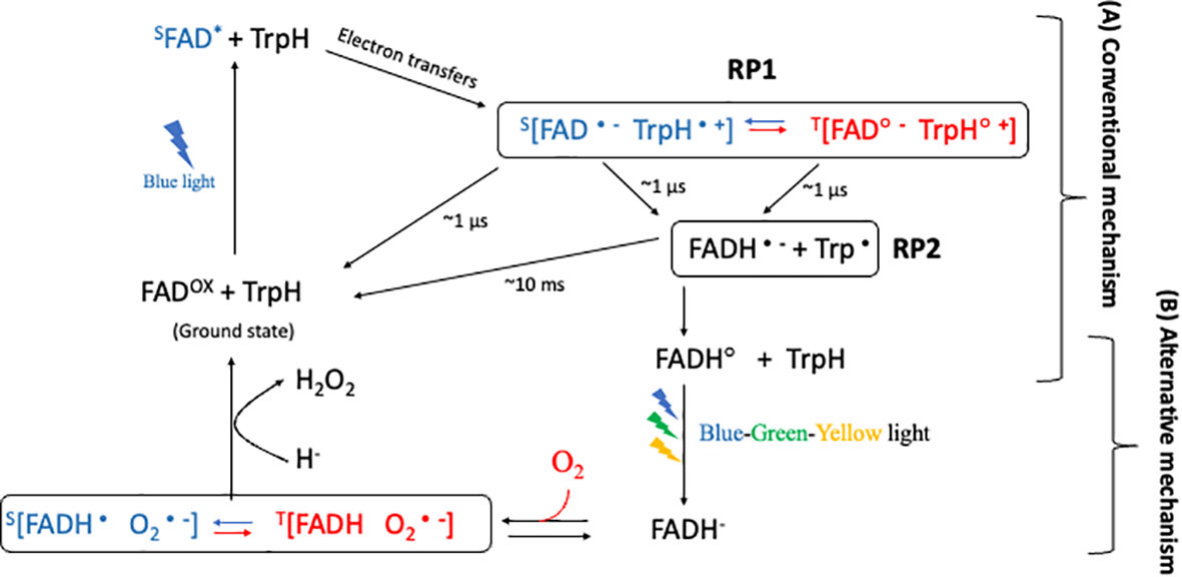
Conventional and Alternative radical-pair mechanism in cryptochrome. After blue-light illumination, the first radical pair, RP1, is generated, with a microsecond-scale lifetime. RP1 is proposed to be the magnetically sensitive radical pair in **(A)** the conventional mechanism. The blue color represents the singlet product, and the red color represents the triplet product during the interconversion between singlet and triplet product. RP2, which is generated from RP1. Subsequent illumination with blue-green-yellow light causes flavin to fully reduce (to FADH-) in the triplet state. In this state, the fully reduced flavin can revert back to the ground state in the presence of oxygen in the dark, generating a superoxide radical pair. The radical [FADH• O2•−] pair, which has a lifetime of several minutes, is therefore suggested to be the magnetically sensitive radical in **(B)** an alternative mechanism.

RPM presents a compelling model for comprehending magnetoreception in cryptochromes, a process contingent upon the formation of specific radical pairs and their prospective sensitivity to magnetic fields. Despite extensive research focusing on this mechanism, uncertainty remains concerning the radical pairs most critical for magnetic sensitivity within the cryptochrome photocycle. Two primary radical-pair hypotheses, namely the conventional and alternative radical-pair mechanisms (**Figure 3**), have emerged, each proposing distinct radical pairs as the primary magnetically sensitive entity (Player and Hore, 2019).

In order to elucidate this, we offered a comprehensive explanation of the photocycle of plant cryptochromes. In this context (Player and Hore, 2019), two radical pairs, termed conventional mechanism (**Figure 3A**) and alternative mechanism (**Figure 3B**), were identified that fulfil some, if not all, the criteria of the radical-pair hypothesis. The *conventional mechanism* emerges from the forward photochemical cycle and necessitates an FADox ground state, wherein the photoreduction of FAD produces a [FAD•− TrpH•+] radical pair. Conversely, the *alternative mechanism* relies on flavin reoxidation and requires the creation of FADH− via either of two methods: light-dependent photoreduction of FADH•, or light-independent reduction through cellular processes. The ensuing reactions between FADH− and oxygen give rise to a semiquinone-superoxide [FADH• O2•−] radical pair.

Consequently, the RPM, both conventional and alternative, offer distinct methods for examining and understanding cryptochrome magnetoreception. However, the intricacy of the photocycles implicated in these mechanisms, coupled with the variability among different cryptochrome types, implies that a comprehensive understanding of magnetoreception will necessitate continued investigation and multifaceted research approaches (Karki et al., 2021).

### 3.5 The conventional radical-pair mechanism: quantum pioneer in the magnetoreception

This mechanism (**Figure 3A**) posits that the crucial step in magnetoreception involves the photoreduction reaction of the cryptochrome photocycle, culminating in the formation of radical pairs. This process generally encompasses the involvement of a photoactivated molecule, often a protein, and an adjacent electron donor, together constituting the radical pair. The FAD and Trp are regularly proposed as the radical pair constituents within cryptochromes. Upon light absorption, the cryptochrome-embedded FAD molecule is excited and accepts an electron from a neighbouring Trp residue, leading to the formation of the radical pair [FAD•− TrpH•+; RP1]. In this radical pair, the unpaired electron spins on the FAD and Trp radicals can assume either a triplet or singlet configuration. The quantum entanglement and quantum coherence of these spins enables the radical pair to subsist in a superposition of these states, with the local magnetic field influencing the interconversion between the singlet and triplet states (Ritz et al., 2010). Additionally, the radicals in the singlet state can revert to the ground state [FAD and TrpH]. However, the radicals in both singlet and triplet states can also advance and become [FADH•Trp•; RP2], which could be referred to as RP2 (Müller et al., 2015; Nohr et al., 2016). The lifetime of RP1 and RP2 is approximately 1 μs and 10 ms, respectively (Maeda et al., 2012). The RP2 appears to have a longer lifetime than RP1 and is more suitable as the potential radical pair for magnetoreception. Nevertheless, the quantum yields of FAD•− in RP1 has strongly anisotropic 14N, therefore, RP1 is presumed to be determined by the Earth’s magnetic fields and can be the basis for magnetic sensing in migratory birds for this mechanism more than RP2 (Solov’yov et al., 2007; Hore and Mouritsen, 2016). In support of this mechanism, several experiments also demonstrated the effects of the magnetic field on forwarding electron transfer of cryptochrome’s (Maeda et al., 2012; Mouritsen and Hore, 2012; Dodson et al., 2013; Sheppard et al., 2017). However, most of the experiments supporting this mechanism are *in-vitro* experiments. For instance, the finding supports the light-dependent RPM postulate that light triggers the formation of radical pairs (Maeda et al., 2012). The presence of a magnetic field influenced these radicals, enhancing the recovery of the FAD ground state, which aligns with the theoretical predictions of RPM. Moreover, the absorption signal of AtCry1 below 420 nm decayed rapidly due to the protonation of FAD•− to FADH•, further underscoring the light-dependent RPM (Maeda et al., 2012). These *in vitro* studies on AtCry1 unambiguously substantiate the conventional light-dependent mechanism of RPM (Maeda et al., 2012).

In addition, the application of RF at the Larmor frequency has emerged as a promising tool for characterizing the properties of the magnetically-sensitive radical pair. Hore and Mouritsen (2016) conducted calculations suggesting that if RF at 1.4 MHz disrupts the orientation of migratory birds, one of the radicals in the pair should be devoid of hyperfine interactions. To explore this, they simulated a system of radical-pair reaction in which the first radical had a strong hyperfine interaction. For the second radical, they varied the number of hyperfine interactions from 0-4. Their findings suggested that if both radicals have several hyperfine interactions, the effect of RF should manifest at a variety of different frequencies. However, if one of the radicals has only one or no hyperfine interaction, the RF effect could be prominent at only the appropriate frequencies. Especially if one radical has no hyperfine interaction, the sensitive effect could present only at 1.4 MHz, the same frequency that showed the effect in the behavioural experiment. In the context of the conventional radical-pair mechanism, the [FAD-Trp] pair aligns with Hore & Mouritsen’s model in which both radicals have significant hyperfine interactions with the nuclei more than 10. Thus, Lee et al. (2014) proposed that this pair might not be sensitive to the stimulation of RF. Furthermore, Deviers et al. (2022) demonstrated that the radical triad system, as previously mentioned, could offer enhanced magnetosensitivity, which seems irreconcilable with the classical RPM, suggesting a shift in our understanding of the mechanism involved in cryptochrome’s magnetoreception (Deviers et al., 2022). This has profound implications for our comprehension of cryptochrome function and its potential role in magnetoreception.

While the existing evidence offers substantial support for the light-dependent RPM, the majority of these insights are derived from *in vitro* studies. This leaves a critical gap in our understanding as *in vivo* validation of this mechanism has yet to be conclusively demonstrated. The complex interplay of biological and environmental factors in a living organism could significantly influence the operation of the RPM. Thus, comprehensive *in vivo* studies are indispensable for fully corroborating the light-dependent RPM and establishing its relevance in real-world biological contexts.

### 3.6 The alternative radical-pair mechanism: the unsuspected *in-vivo* triumph

Contrary to the conventional mechanism, the alternative radical-pair mechanism posits the radical pair [FADH• O2•−] as the principal magnetosensing candidate (Ahmad, 2016). Lee et al., (2014) postulated that this pair might exhibit greater sensitivity to Earth’s magnetic fields than the [FAD•− TrpH•+] pair. This radical pair endures for several minutes (Herbel et al., 2013), a duration suitable for magnetic interaction, an idea supported by numerous studies (Maeda et al., 2008; Ritz et al., 2009; Müller and Ahmad, 2011).

However, despite the extended lifespan of the [FADH• O2•−] pair, the spin-relaxation time of the superoxide radical is exceptionally short, which could impede its potential as a magnetically sensitive molecule (Karogodina et al., 2009; Karogodina et al., 2011; Kattnig and Hore, 2017). To counteract the rapid spin relaxation issue, Kattnig and Hore (2017) suggested an enhanced model of the radical-pair mechanism incorporating a radical scavenging system. Nevertheless, this model remains theoretical, awaiting empirical validation. Recently, Hong and Pachter (2023) conducted spin dynamics simulations to investigate the influence of inter-radical interactions and scavenging radicals on magneto-sensitivity. They explored various radical pairs and examined how the presence of scavenging radicals affects the spin dynamics of these pairs. Through their simulations, they assessed the impact of scavenging radicals on the spin relaxation rates and overall magneto-sensitivity of the radical pairs. By analyzing the spin dynamics of the proposed radical pairs and considering the effects of scavenging radicals, Hong and Pachter (2023) offer valuable insights into the mechanisms that could enhance magneto-sensitivity. Their theoretical framework provides an understanding of the interplay between inter-radical interactions and scavenging radicals, which could potentially address the rapid spin relaxation issue in the enhanced model proposed by (Kattnig and Hore, 2017). It is important to note that the study by Hong and Pachter (2023) is based on theoretical spin dynamics simulations. While their findings are valuable, further empirical validation is necessary to confirm the effectiveness of the proposed mechanisms. Therefore, their paper serves as a foundation for future experimental studies to investigate the role of scavenging radicals in enhancing magneto-sensitivity and addressing the rapid spin relaxation issue in the radical-pair mechanism. Intriguingly, a more recent study by Deviers et al. (2022) proposed a radical triad system, consisting of the flavin semiquinone, the superoxide, and a tyrosine or ascorbyl scavenger radical, could provide superior magnetosensitivity under realistic conditions. The spin-selective recombination reaction with a third radical in this system may enhance magnetosensitivity, offering new insights into the RPM and the potential role of cryptochrome in light-independent magnetoreception (Deviers et al., 2022). Player and Hore (2019) demonstrated that specific restrictive conditions are necessary to mitigate the rapid relaxation time of superoxide. To address the problem of rapid spin relaxation time, as discussed by Kattnig and Hore (2017), several strategies can be explored. One approach is the chemical control of spin-lattice relaxation in molecular qubits, as demonstrated by Amdur et al., (2022). By understanding and manipulating molecular vibrations and relaxation times, it may be possible to enhance spin relaxation time in superoxide-containing radical pairs. Amdur et al., (2022) investigated this control to discover a room temperature molecular qubit, highlighting the potential of molecular electronic spins as a novel platform for qubit structure and control. Additionally, the study by Nicolaides et al. (2023) provides insights into spin pumping from organic radical films, presenting another avenue to explore. They demonstrated spin current emission from a Blatter-type radical with outstanding stability and low roughness, indicating the possibility of realizing spin pumping even in the absence of long-range ferromagnetic order (Nicolaides et al., 2023). These approaches, along with the deposition of highly-ordered arrays on solid surfaces to create 2D-lattices and doping with controlled amounts of spin-free radicals, can contribute to solving the rapid spin relaxation issue and foster the development of suitable molecular qubits with potential industrial applications (Xu and Ping, 2023).

Additionally, some *in-vivo* studies also reported results supporting the alternative mechanism of RPM. For instance, some reports demonstrated that migratory birds could also orient to Earth’s magnetic direction in green light at about 565 nm. In this case, the flavin cofactor of cryptochrome should be able to absorb the light at 565 nm (Green light). One possible flavin state that can absorb light at around 565 nm is the neutral radical redox FADH° state of cryptochrome, which reduces to FADH- after green light absorption (Nießner et al., 2013; Nießner et al., 2014). From this state, there is no possibility for the radical pair [FAD•− TrpH•+] to form and interact with magnetic fields. Additionally, from the behavioural experiment where birds were exposed to an intermittent light/dark cycle and a magnetic field present only in the dark interval, birds were found to orient in the magnetic direction, even when the magnetic field exposure was only during the dark period (reoxidation step). These findings support the radical-pair that formed during flavin reoxidation of cryptochrome in the dark as the possible magnetically-sensitive radical-pair for magnetoreception, known as the alternative radical-pair mechanism (Wiltschko et al., 2016).

Interestingly, in the plant model, results from Xu et al. (2014) showed the effect of magnetic fields on the dephosphorylation of *Arabidopsis* cryptochrome in darkness. Also, Agliassa et al. (2018b) demonstrated the impact of geomagnetic fields on the photomorphogenic-promoting gene expression in etiolated seedlings, which they linked to the light-independent mechanism. Recently, similar results from Agliassa and Maffei (2019) reported the expression of plant endogenous clock genes that was altered after exposure to the near-null field in the dark condition (Agliassa and Maffei, 2019). These magnetically responsive results in the dark imply that the reoxidation step might be the magnetically-sensitive reaction in the cryptochrome photocycle. Therefore, the potential magnetic sensitivity radical-pair for this mechanism should be [FADH- O2•−].

Significantly, our own laboratory work has been instrumental in advancing our understanding of the alternative RPM. Our research with *Arabidopsis* cryptochrome-1, as presented in Pooam et al. (2019) and Hammad et al. (2020), offers compelling evidence for the role of magnetic fields in cryptochrome activity. We have observed that exposure to a static magnetic field can enhance cryptochrome responses to light, a phenomenon that is especially noticeable when the magnetic field is applied during dark intervals between light exposures. These findings suggest that flavin reoxidation, potentially involving reactive oxygen species (Procopio et al., 2016), could be the magnetically sensitive step in the cryptochrome photocycle. This alternative mechanism contrasts with the traditional focus on the forward electron transfer reaction involving Trp• and Tyr• radicals (Mouritsen and Hore, 2012; Dodson et al., 2013; Hong and Pachter, 2015; Lüdemann et al., 2015; Worster et al., 2016) and instead emphasises the importance of dark-state reactions.

Our research results suggest a shift in the narrative surrounding cryptochromes and their role in magnetosensing. We have uncovered the significance of the flavin reoxidation reaction and its interaction with a magnetic field in the *Arabidopsis* cryptochrome photocycle, a subject thoroughly reviewed (Chaves et al., 2011; Ahmad, 2016b; Hammad et al., 2020). Our comprehension of the cryptochrome photocycle has evolved to recognise that the flavin reoxidation – the conversion of FADH• back to FADox – plays a pivotal role in cryptochrome magneto-sensitivity. This conversion reaction, likely involving the superoxide radical (O2•−), is susceptible to magnetic field effects, thereby introducing a new pathway for magneto-sensitivity in cryptochromes. This perspective significantly expands our understanding of the cryptochrome photocycle and its role in magnetosensing, highlighting that much remains to be learnt about the exact mechanisms at play.

The recent study by Deviers et al. (2022) lends credence to this hypothesis, demonstrating that the reoxidation reaction of the reduced FAD cofactor in cryptochrome, potentially involving a radical triad system, can display remarkable magnetosensitivity under realistic conditions. This study thus reinforces the concept that the radical triad system, comprising the flavin semiquinone, the superoxide, and a scavenger radical, might play a crucial role in cryptochrome’s magnetosensitivity (Deviers et al., 2022). Our understanding of the radical-pair mechanism and its role in magnetoreception continues to evolve. The long-established model of the [FAD•− TrpH•+] radical pair serving as the primary magnetosensing agent is being contested by newer findings that highlight the potential of the [FADH• O2•−] pair, and even more so the radical triad system (Deviers et al., 2022).

Our own research has contributed to this shifting perspective, shedding light on the possibility that the flavin reoxidation reaction, involving the [FADH• O2•−] pair or a triad system, could be a magnetically sensitive step in the cryptochrome photocycle. This expanded understanding could have profound implications for our understanding of cryptochrome activity, magnetoreception, and, by extension, navigation in migratory animals.

Although the light-independent variant of the RPM provides a promising alternative for explaining magnetoreception in scenarios like plant growth where light may not be readily available, it also faces several challenges and limitations, inherently tied to the complexities of quantum mechanics and the nature of the radicals involved.

Firstly, as with the light-dependent RPM, the light-independent variant faces the challenge of maintaining quantum coherence in biological systems. Decoherence – the loss of quantum coherence – is a ubiquitous problem in quantum systems and is thought to occur rapidly in the warm, wet, and noisy environment of biological cells (Schlosshauer, 2007). A crucial question is how long quantum coherence must be maintained in radical pairs to influence biological processes. Current research suggests that the timescales could be surprisingly long, requiring mechanisms to protect the coherence (Hore and Mouritsen, 2016). Moreover, our theoretical understanding of how magnetic fields could influence the recombination rates or product yields of radical pairs remains underdeveloped. Current quantum mechanical models, although useful, do not provide comprehensive insight into these effects, particularly for complex biological systems with many interacting components (Kominis, 2009).

Secondly, this light-independent RPM proposes the involvement of biologically ubiquitous, yet highly reactive molecules like superoxide radicals. Superoxide, a byproduct of cellular metabolism, is suggested to form radical pairs whose spin states could be influenced by magnetic fields, facilitating magnetoreception (Buchachenko, 2016). However, superoxide is a potent ROS, and its levels are tightly regulated within the cell due to its potential to cause oxidative damage. How organisms might harness superoxide for magnetoreception without disturbing the delicate redox balance is a significant open question. Yet, we believe that the level of ROS production from the cryptochrome photocycle is at a mild level and might not disrupt the redox balance.

Thus, while the light-independent RPM opens new avenues for understanding magnetoreception, it also poses significant challenges. The interplay of quantum coherence, the role of radicals like superoxide, and the theoretical modelling of radical pair dynamics under magnetic fields represent key areas for future research.

In conclusion, both the conventional and alternative RPMs have their merits and limitations, but the alternative RPM provides a compelling avenue for future research. Further studies are needed to expand our understanding of this complex biological phenomenon, potentially leading to novel insights into magnetoreception in plants and other organisms.

### 3.7 Quantum yield in conventional and alternative mechanisms of radical pair formation in magnetoreception

In the realm of magnetoreception, the quantum yield of radical pairs holds significant importance. Quantum yield refers to the efficiency of generating radicals through a photochemical process, with higher values indicating greater efficiency (Hiscock et al., 2016). Several studies have delved into the quantum yield of radical pairs in plant cryptochrome, such as [FAD•− TrpH•+], [FADH•Trp•], and [FADH• O2•−], revealing their magnetic sensitivity (Maeda et al., 2012; Müller et al., 2014; Procopio et al., 2016).

For instance, Maeda et al. (2012) conducted a significant study on the kinetics and quantum yields of photo-induced flavin-tryptophan radical pairs in cryptochrome, finding them to be magnetically sensitive. The research clarified the mechanistic origin of the magnetic field effect and determined the rates of various processes involved. While the study indicates that the quantum yield of radical pairs in plant cryptochrome can be influenced by the magnetic field, it does not provide specific quantum yield values for different states or the exact value for the formation of a secondary species (RP2) from the magnetically sensitive radical pair RP1 in the conventional mechanism. The findings suggest an influence of the magnetic field on quantum yield but leave room for further exploration to pinpoint exact values. Furthermore, Müller et al. (2014) estimated the quantum yield of the FADH· Trp· radical pair formation in isolated Arabidopsis cryptochrome 1 (At CRY1) to be approximately 2%. Procopio et al. (2016) further linked the quantum yield of FADH° formation to the biological function of plant cryptochrome. However, the specific quantum yields of each radical pair and their modulation by the magnetic field are often not directly provided. This gap in knowledge extends to the probability of simultaneous radical formation in cryptochromes, a complex issue that warrants further exploration.

In the context of the Alternative Mechanism, the quantum yield of radical pair formation should be lower at certain light intensities, reflecting its formation from the radical pair in the Conventional Mechanism. This aspect, along with the probability that two or three radicals are formed simultaneously in all cryptochromes in a plant cell, presents intriguing questions for future research.

Understanding the quantum yield of radical pair formation in magnetoreception, particularly in the Conventional and Alternative Mechanisms, is an intricate and essential aspect that warrants further exploration. The existing studies, while providing valuable insights, leave certain questions unanswered, such as the specific quantum yield values for various radical pairs and the probability of simultaneous radical formation in cryptochromes.

Future research could focus on designing controlled experiments to measure the quantum yield of different radical pairs at varying light intensities. By utilizing advanced spectroscopic techniques and computational modeling, researchers could investigate the formation and interconversion of radical pairs in both the Conventional and Alternative Mechanisms. Special attention could be given to understanding how the quantum yield of radical pair formation in the Alternative Mechanism might be lower than that in the Conventional Mechanism at certain light intensities. Additionally, the study of the triad system and the probability that two or three radicals are formed simultaneously in all cryptochromes in a plant cell could be explored through targeted experiments. This might involve the development of new methodologies or the adaptation of existing techniques to specifically probe these aspects. Furthermore, collaboration between experimentalists and theorists could foster a more comprehensive understanding of how magnetic fields influence the quantum yield of radical pairs. This interdisciplinary approach could lead to the development of new theoretical models that more accurately represent the complex dynamics of magnetoreception.

Thus, the quantum yield of radical pair formation in magnetoreception presents a promising and rich area for further investigation. By focusing on the specific aspects mentioned above and employing a combination of experimental and theoretical approaches, researchers could unravel new dimensions in the study of magnetoreception. This exploration could not only enhance our understanding of this complex biological phenomenon but also pave the way for innovative applications in various fields.

### 3.8 Cracking the code of cryptochrome: advanced approaches and future directions in plant magnetoreception research

Evidence from some studies have already confirmed that the light-independent reaction of cryptochrome is a crucial step for magnetoreception. Nonetheless, further corroboration from independent laboratories, and the application of diverse experimental techniques, could be essential for the validation of this mechanism. Therefore, the fascinating field of plant magnetoreception presents several opportunities for future experimental studies. These could potentially resolve the ongoing debate between the conventional and alternative radical-pair mechanisms. Here are some research directions that plant biologists might contemplate.

Initially, investigations into the structure and dynamics of cryptochromes could be of great value. Structural investigations, such as those employing X-ray crystallography or cryo-electron microscopy, might provide critical insights into the binding of superoxide and other radicals to cryptochromes (Brautigam et al., 2004). This could help to determine whether the alternative RPM has a structural foundation. Molecular dynamics simulations could supplement these structural investigations by providing a dynamic perspective on radical interactions within the cryptochrome (Solov’yov and Schulten, 2012). These structural techniques could furnish us with precise, high-resolution images of the radical (superoxide, for instance) binding to the cryptochrome. If the radicals bind and interact in a way that is consistent with the conventional RPM, then that model is likely accurate. If the interaction aligns more with the alternative RPM, then that model might be the valid one.

Moreover, another promising starting point is absorption spectroscopy. This method can illuminate the potential light-dependent behavior of the cryptochrome photocycle. By subjecting cryptochrome to light of various wavelengths and intensities and observing the resultant absorption spectra, we can gain insights into the photocycle’s response to light. In the conventional RPM, the formation and recombination of radical pairs are directly linked to the absorption of light. Therefore, if we observe changes in the absorption spectra that align with the predicted behavior of the radical pairs under this model, that will provide evidence supporting the conventional RPM. For instance, we might see absorption peaks corresponding to the energy differences between the singlet and triplet states of the radical pairs. Conversely, if the alternative RPM involves additional light-triggered steps or a different mechanism, we might expect to see different patterns in the absorption spectra. For example, there might be additional absorption peaks or variations in peak intensities that cannot be explained by the conventional RPM. To unravel the temporal dynamics of this response, one could consider using pulsed light instead of continuous light. Conversely, to probe the light-independent reactions, a parallel set of observations could be carried out under complete darkness, providing a contrasting backdrop to the light conditions (Ozturk, 2017). Complementing these experiments, electron paramagnetic resonance (EPR) spectroscopy could be employed to detect the presence of radical pairs within cryptochrome under different light conditions. This technique, capable of detecting unpaired electron spins, offers a direct probe into the RPM (Sheppard et al., 2017).

Additionally, *in vivo* studies using plants could be conducted. For instance, genetically engineering plants with altered cryptochrome structures could help reveal the critical components for magnetoreception. Genetic manipulation techniques, such as CRISPR/Cas9, could be employed to create these modified plants (Ma et al., 2015). Cryptochrome, the protein hypothesized to mediate magnetic sensing, has a specific structure. Certain parts of this structure are thought to be critical for its function. Using genetic engineering tools like CRISPR/Cas9, we could alter the structure of cryptochrome in the plants. These alterations could target the specific parts of cryptochrome that are believed to be important for its magnetic sensing function. If the conventional RPM is correct, then these parts of the cryptochrome structure would interact with radicals in a particular way to sense magnetic fields. The conventional RPM suggests that the radical pair is formed between a FAD molecule, which is the chromophore of the cryptochrome, and a Trp residue (Trp-triad) within the protein structure. In the alternative RPM, it’s suggested that a superoxide radical, or another similar radical, binds to the FAD molecule to form the radical pair. This would mean that the binding site for this radical would be located on the FAD molecule. In terms of genetic engineering, it might be possible to modify the Trp residues in the Trp-triad, or the binding site on the FAD molecule, to test the predictions of the conventional and alternative RPMs. Nevertheless, this would require a highly detailed understanding of the cryptochrome structure and function, and the ability to make precise modifications to the protein sequence. To date, the specific site of each radical pair in the cryptochrome structure is not clearly understood.

Finally, it might be worthwhile exploring the potential influence of other environmental factors on magnetoreception. Could variables such as temperature or light intensity influence the efficiency of magnetoreception? Answering these questions could shed light on whether the conventional or alternative RPM is more viable under different environmental conditions. For example, in the case of temperature, Temperature effects on singlet-triplet interconversion: In the context of the RPM, a key feature of radical pairs is their ability to interconvert between singlet and triplet states, which is a quantum mechanical process that can be influenced by magnetic fields. The rates of singlet-triplet interconversion could potentially also be influenced by temperature, although the details of this are likely to be complex and could depend on specific characteristics of the radical pair and its environment.

A notable reference discussing the impact of temperature on the RPM in birds is a paper by Henrik Mouritsen and colleagues (Mouritsen et al., 2016). In this study, the authors argue that the key radical pair in avian cryptochrome (FADH•-TrpH•+) is indeed temperature-sensitive, which could provide an explanation for the experimentally observed effects of temperature on bird magnetoreception. To definitively determine the effects of temperature on magnetoreception, and to help differentiate between the conventional and alternative RPMs, one could design experiments in which the temperature is systematically varied while monitoring magnetoreception. Comparing the experimental results to the predictions of the conventional and alternative RPMs, taking into account their respective expectations for temperature effects, could provide further insights into which model is more likely to be correct. However, it’s important to note that interpreting these experiments may be challenging, due to the complex and potentially varied ways in which temperature can influence radical pair dynamics.

In the case of light, the conventional and alternative RPMs, both would theoretically be affected by light intensity, but potentially in different ways. For the conventional RPM, light is necessary to form the radical pair. Therefore, under low light conditions, the formation of radical pairs might be limited, potentially reducing the strength or reliability of the magnetoreception signal. Conversely, under high light conditions, more radical pairs would be expected to form, possibly enhancing magnetoreception. For the alternative RPM, if it involves some sort of light-triggered enzymatic process or additional light-sensitive step, then its dependence on light intensity might be more complicated. For example, there might be an optimal light intensity that maximizes the magnetoreception signal, with either too little or too much light reducing efficiency. By conducting experiments with varying light intensities, we could compare the resulting magnetoreception abilities to the predictions made by each RPM. For instance, if we find that magnetoreception abilities increase linearly with light intensity, this might support the conventional RPM. Alternatively, if we observe a more complex relationship between light intensity and magnetoreception, this could provide evidence for the alternative RPM.

Each of these suggested experiments could potentially provide critical insights into the mechanisms of plant magnetoreception. They each represent a piece of a larger puzzle, and together, they could lead us towards a more comprehensive understanding of this intriguing biological phenomenon. Nevertheless, experiment on plant magnetoreception frequently yields inconsistent results that prove challenging to replicate. Such inconsistencies might originate from the intricate dynamical degrees of freedom influencing magnetic compass sensitivity in cryptochromes, as depicted by the recent study by Grüning et al. (2022). In their groundbreaking paper, Grüning et al. (2022) examined the magnetic compass sensitivity of cryptochromes from plants (*Arabidopsis* thaliana AtCry1) and avian species (European robin Erithacus rubecula ErCry4a, and pigeon Columba livia ClCry4a). The researchers utilized molecular dynamics simulations and density functional theory-derived hyperfine interactions to scrutinize the impact of thermal motion and fluctuations in the dihedral and librational angles of FAD and Trp radicals on radical pair recombination dependent on the geomagnetic field direction. Their findings disclose that librational motions of TrpC•+ in avian cryptochromes are considerably smaller than in AtCry1. This suggests that the variations in the dynamical properties of cryptochromes across species might contribute to the inconsistent results frequently observed in plant magnetoreception studies. Additionally, they demonstrated that the magnetic sensitivity of cryptochromes isn’t solely dependent on singular snapshots of protein structure, but should also consider the ensemble properties of hyperfine interactions. This revelation provides insight into why research in this field often produces inconsistent and hard-to-replicate results.

In conclusion, the validation of the magnetoreception mechanism in plants necessitates rigorous multidisciplinary investigation. Studies must delve into cryptochrome structures, their light-dependent behavior, and the impact of environmental factors. Further, the inconsistencies often seen in magnetoreception research underscore the complexity of this biological process. By exploring these proposed research directions, we may garner insights into this fascinating phenomenon, ultimately clarifying the underpinnings of plant magnetoreception.

## 4 Beyond boundaries: exploring magnetoreception’s hidden dimensions:

While the RPM has led magnetoreception research, new theories emerge. LCM proposes that magnetic fields affect molecular energy transitions, potentially explaining plant magnetoreception. Meanwhile, the Magnetite-based theory suggests the protein complex MagR and biogenic magnetite offer insights into non-avian magnetoreception. These fresh perspectives could revolutionize our understanding of how organisms navigate Earth’s magnetic field.

### 4.1 Level crossing mechanism: the quantum vanguard unraveling magnetoreception’s mysteries

The LCM is a budding quantum-based theory of magnetoreception offering an engaging alternative to the RPM (Binhi, 2019). Contrary to the RPM’s assertion that magnetic fields modulate radical pair-involved reactions, the LCM posits that these fields could influence transitions between energy levels of magnetic moments in molecules or atoms (Binhi, 2019).

At its core, the LCM is based on a principle of quantum mechanics known as “level crossing,” a phenomenon where the energies of two states within a quantum system become identical under the influence of an external field, leading to the states’ merging. In the context of magnetoreception, this external field is the geomagnetic field, and the quantum system may be represented by certain magnetic molecules or atoms within a biological entity. This suggests that changes in the geomagnetic field could instigate level crossings, thus influencing biological processes (Binhi, 2016).

To better grasp this concept, visualize a busy train station with each train symbolizing different energy levels of magnetic moments in molecules or atoms. Each train travels at a distinct speed, indicating different energy states. Sometimes, two trains (energy levels) move side by side at the same pace, a brief synchronization akin to the “level crossing” in quantum mechanics. The stationmaster, representing the geomagnetic field, can alter the trains’ speeds by manipulating the track switches. As the stationmaster alters these tracks, trains initially moving at different speeds might end up side by side, their speeds synchronized, thereby creating a “level crossing.” In this metaphor, the brief synchronization of the trains’ speeds symbolizes the energy levels of magnetic moments in molecules or atoms within a biological system becoming identical under the geomagnetic field’s influence. These changes in the trains’ speeds and routes could then affect the passengers (biological processes), potentially inciting changes in their behaviors or conditions. Just like the stationmaster’s ability to influence the trains’ speeds and paths, causing occasional synchronization that impacts passengers, the geomagnetic field can affect certain magnetic molecules or atoms’ energy levels, potentially influencing biological processes.

Experimental evidence supporting the LCM as a feasible magnetoreception mechanism stems from studies on the model plant organism, *Arabidopsis thaliana*. Researchers noted magnetic effects in various processes like cryptochrome- and blue light-independent seed germination, red-light elicited anthocyanin accumulation, and hypocotyl elongation in darkness (Agliassa et al., 2018b; Dhiman and Galland, 2018) These phenomena could not be adequately explained by the cryptochrome-mediated RPM, thereby aligning more closely with the LCM’s predictions, which anticipate multiple maxima in stimulus-response curves and the effects of magnetic field reversals (Binhi, 2019).

A thorough investigation by Dhiman et al. (2023) further underscored the potential relevance of the LCM in understanding magnetoreception. The researchers scrutinized the magnetoreception of *Arabidopsis thaliana* by analyzing several developmental responses in weak static magnetic fields. The study determined that a magnetic field of 50 μT could accelerate seed germination by approximately 20 hours, consistent across the wild-type strain and the cry1cry2 double mutant, which lacks cryptochromes 1 and 2 (Dhiman et al., 2023).

Interestingly, the study revealed that the magnetic field-induced germination acceleration was obscured in the presence of exogenous sucrose. In addition, the researchers identified maxima near 9 and 60 μT in the stimulus-response curves for hypocotyl elongation. Moreover, the photo-accumulation of anthocyanins and chlorophylls could also be modulated effectively by magnetic fields when exposed to low-irradiance red and blue light, respectively (Dhiman et al., 2023).

These findings suggest the existence of light-independent mechanisms of magnetic field reception in *Arabidopsis thaliana*, mechanisms that remain unidentified. Furthermore, the study outcomes align more closely with the LCM’s predictions than those of the cryptochrome-associated RPM. This alignment provides additional support for the potential significance and applicability of the LCM in explaining magnetoreception phenomena in plants (Dhiman et al., 2023).

In summary, while the LCM’s capacity to explain the intricate responses of biological systems to magnetic fields suggests a substantial role in our understanding of magnetoreception, further research is required. This research aims to fully explore and validate the LCM’s implications, especially concerning the established RPM and cryptochrome-mediated processes in plants. However, it is crucial to recognize that the LCM, as a relatively new theory in the field of magnetoreception, has certain limitations and challenges that have yet to be fully addressed.

One of the key limitations of the LCM is the absence of direct experimental evidence. While studies such as those conducted by Dhiman et al. (2023) and Agliassa et al. (2018b) have yielded results aligned with LCM predictions, they do not conclusively demonstrate the operation of the LCM in magnetoreception. The observed responses could potentially be explained by other, as-yet-unknown mechanisms. Thus, while the LCM can explain certain observations, direct proof of its operation in biological organisms remains elusive.

Another limitation relates to the identification of molecular candidates that can undergo level crossing under the Earth’s magnetic field. As of 2021, no specific molecule or atomic structure has been identified in biological organisms that could serve this function. This stands in contrast with the RPM, where cryptochromes have been identified as potential magnetoreceptors (Ritz et al., 2000).

Moreover, maintaining quantum coherence in a biological environment, a significant challenge for any quantum-based theory, is of particular concern for the LCM. Quantum coherence refers to the maintenance of the phase relationship between different states in a quantum superposition, a key requirement for quantum phenomena like level crossing to occur. However, biological systems are typically warm and wet, conditions that can cause rapid decoherence, disrupting the delicate quantum states (Schlosshauer, 2007).

Lastly, the LCM, like any scientific theory, is subject to change and refinement as new evidence becomes available. Currently, the LCM is a promising but not yet fully established theory of magnetoreception. Further empirical research is needed to validate and potentially expand upon this theory.

Building upon the LCM as a plausible theory for plant magnetoreception necessitates rigorous experimentation (Binhi, 2019). An ideal starting point is investigating the LCM’s prediction of a resonant response to specific frequencies of magnetic field oscillation, although exact frequencies are yet to be identified. These can be experimentally determined, with plants subjected to a range of frequencies and subsequent changes in biological processes, such as seed germination or root growth direction, being meticulously recorded. If particular frequencies induce substantial effects, it would corroborate the LCM (Binhi, 2019).

Another intriguing aspect of the LCM is the requirement of quantum coherence (Schlosshauer, 2007). Quantum coherence, in the context of the LCM, refers to the maintenance of specific phase relationships between quantum states in a superposition, enabling the system to exhibit behaviors uniquely quantum mechanical in nature. Detecting quantum coherence under biological conditions, therefore, is a significant step in affirming the LCM. Techniques such as electron spin resonance or quantum beat spectroscopy, capable of detecting unpaired electron spins or interference patterns in fluorescence following excitation respectively, could be employed for this purpose (Lambert et al., 2012). If quantum coherence can be convincingly demonstrated in plant samples, it would further reinforce the LCM’s standing.

Lastly, the LCM predicts a distinct temperature dependence compared to the RPM (Binhi, 2019). To understand why temperature could be a significant factor, it’s crucial to realize that both the LCM and the RPM are quantum mechanical processes, and temperature can influence quantum states. In the case of the LCM, magnetic field influences can cause certain energy levels in a system to cross, leading to changes in the system’s behavior that could affect biological processes. How much these levels cross, and the biological consequences of these crossings, could well depend on temperature. However, the specific temperature points that would validate the LCM are not yet clear. This area requires further theoretical development and experimental testing. An experimental approach might involve subjecting plants to a range of different temperatures, then measuring their responses to magnetic fields under these different conditions. If the changes in responses at certain temperatures align with the predictions of the LCM but not the RPM, this could provide evidence to support the LCM. By cultivating plants across a temperature spectrum and monitoring alterations in their magnetic responses, it could be ascertained which model’s predictions better coincide with empirical observations. If the observed temperature dependence matches the LCM’s predictions, it would bolster its acceptance.

In summary, these experimental strategies, focusing on frequency specificity, quantum coherence, and temperature dependence, provide promising paths to validate and refine the LCM, thereby advancing our understanding of plant magnetoreception.

### 4.2 MagR: magnetite’s maverick transitioning from foe to ally in magnetoreception

In the captivating realm of magnetoreception, two predominant theories have garnered significant attention: the RPM, focusing on cryptochromes that have been previously discussed, and the Magnetite-based theory, which hones in on the protein complex MagR (Kirschvink and Gould, 1981; Dodson et al., 2013). The Magnetite-based theory postulates that magnetoreception transpires through biogenic magnetite, a ferromagnetic mineral pervasive in numerous organisms, analogous to a natural compass system at the molecular level. The protein complex, MagR, is envisaged as part of this magnetite-based receptor complex, with magnetite crystals within the MagR complex aligning to Earth’s magnetic field, thereby generating a mechanical force that might trigger a biochemical response (Qin et al., 2016). This theory has been implicated in non-avian species such as Drosophila and mice; however, its role in plant magnetoreception is less understood, with cryptochrome-based mechanisms predominantly at the forefront (Ahmad, 2016; Qin et al., 2016).

Parmagnani et al. (2022) have unveiled fresh insights into this complex landscape through their exploration of the formation and function of iron-sulfur (Fe-S) clusters in plants, particularly focusing on how geomagnetic fields could influence them. Their paper posits that GMF reduction affects the amplitude of certain clock genes in *Arabidopsis thaliana*, thereby influencing processes such as plant growth and development, and possibly modulating the expression of various genes. The protein MagR, familiar from the magnetite-based theory, is postulated as a potential biomagnetic sensor in plants due to its capacity to form complexes with other proteins and Fe-S clusters. Furthermore, they hint at a potential integration of MagR and cryptochrome theories, suggesting a complex mechanism that marries elements of light sensitivity and magnetic field alignment (Qin et al., 2016; Xu et al., 2018). In this model, cryptochrome serves as a light sensor, inducing a conformational change in the MagR protein upon activation, which could alter the orientation of the embedded magnetite crystals within the MagR complex. The involvement of the blue-light receptor cryptochrome in magnetic sensitivity is also invoked to support this integrated theory (Parmagnani et al., 2022).

Nevertheless, the empirical evidence buttressing this composite magnetoreceptor is scant, and it remains ambiguous whether the MagR-cryptochrome complex operates via the RPM or a magnetite-based mechanism (Dodson et al., 2013). Despite potential advancements, the MagR theory exhibits certain shortcomings compared to the RPM in the context of plant magnetoreception, primarily due to the more comprehensive understanding and extensive study of cryptochrome-based mechanisms (Ahmad, 2016; Qin et al., 2016; Parmagnani et al., 2022).

In order to solidify the MagR theory, often termed as the ‘Ancient Rival’ of RPM in the realm of magnetoreception, several experimental approaches are possible. We could scrutinize the presence and distribution of magnetite in plants using methods like magnetic force microscopy or transmission electron microscopy. Altering the MagR genes in plants and subjecting them to different magnetic fields under controlled light conditions could shed light on MagR’s impact on magnetoreception. This ‘old adversary’ seems increasingly like a ‘new ally’, potentially contributing to our understanding of magnetoreception in organisms. A sophisticated understanding of the interplay between these two mechanisms—cryptochrome-based and MagR-based—is pivotal for advancing our knowledge of plant magnetoreception.

## 5 Conclusions

In conclusion, this review synthesizes our current knowledge of the magnetoreceptive abilities of plants, underscoring the fascinating interplay between quantum and classical biology. The profound implications of these mechanisms, ranging from influencing plant growth and development to potentially impacting broader agricultural practices (Galland and Pazur, 2005), serve to emphasize the significance of this field of study. As we navigate the labyrinth of plant magnetoreception, we are reminded of the complexity of nature’s adaptations to environmental cues (Brautigam et al., 2004). The exciting prospect of delving further into the relationship between the RPM, LCM, and the MagR theory invites a multi-faceted exploration of the magnetoreception phenomenon. Future research will undoubtedly benefit from adopting an integrative approach, simultaneously probing the individual contributions and interactions of these theories. Moreover, an exciting dimension of this exploration is the potential industrial applications involving cryptochromes. The recent study also provides valuable insights into this area. It explores the use of photogenerated spin-correlated radical pairs (SCRPs) as molecular qubits for quantum sensing, highlighting the potential for resolution-enhanced imaging tools, magnetometers, and magnetic switches. The synthetic tunability in molecular systems offers promising avenues for the design of next-generation selective magneto-sensing devices and molecular qubits for quantum technologies (Mani, 2022).

While there is still a vast expanse of the plant magnetoreception landscape yet to be traversed, the direction provided by this review, coupled with relentless scientific curiosity, promises intriguing discoveries. As we continue to unravel the mysteries of this elusive sense, the doors to understanding how life has evolved to sense and respond to our planet’s magnetic field remain wide open. We are only beginning to scratch the surface, and the journey ahead is teeming with untold promise and potential. Thus, this review does not signify an end but rather serves as an inspiring prelude to the thrilling journey of discovery that lies ahead in the realm of plant magnetoreception, including the innovative industrial applications of cryptochromes in quantum technologies.

## Author contributions

TT: Writing - original draft, Writing - review & editing. KT: Writing - review & editing. KK: Writing - review & editing. LT: Writing - review & editing. ME-E: Writing - review & editing. BA: Writing - review & editing. NJ: Writing - review & editing. KB: Writing - review & editing. MP: Conceptualization, Supervision, Validation, Writing - original draft, Writing - review & editing. 

## References

[B1] Abhary M. Akhkha A. (2023). Effects of neodymium magneto-priming on seed germination and salinity tolerance in tomato. Bioelectromagnetics 44 (1-2), 47–56. doi: 10.1002/bem.22438 36808751

[B2] Agliassa C. Maffei M. E. (2019). Reduction of geomagnetic field (GMF) to near null magnetic field (NNMF) affects some Arabidopsis thaliana clock genes amplitude in a light independent manner. J. Plant Physiol. 232, 23–26. doi: 10.1016/J.JPLPH.2018.11.008 30530200

[B3] Agliassa C. Narayana R. Bertea C. M. Rodgers C. T. Maffei M. E. (2018a). Reduction of the geomagnetic field delays Arabidopsis thaliana flowering time through downregulation of flowering-related genes. Bioelectromagnetics 39, 361–374. doi: 10.1002/BEM.22123 29709075 PMC6032911

[B4] Agliassa C. Narayana R. Christie J. M. Maffei M. E. (2018b). Geomagnetic field impacts on cryptochrome and phytochrome signaling. J. Photochem. Photobiol. B. 185, 32–40. doi: 10.1016/J.JPHOTOBIOL.2018.05.027 29864723

[B5] Ahmad M. (2016). Photocycle and signaling mechanisms of plant cryptochromes. Curr. Opin. Plant Biol. 33, 108–115. doi: 10.1016/J.PBI.2016.06.013 27423124

[B6] Ahmad M. Cashmore A. R. (1993). HY4 gene of A. thaliana encodes a protein with characteristics of a blue-light photoreceptor. Nature 366, 162–166. doi: 10.1038/366162A0 8232555

[B7] Albaqami M. Hammad M. Pooam M. Procopio M. Sameti M. Ritz T.. (2020). Arabidopsis cryptochrome is responsive to radiofrequency (rf) electromagnetic fields. Sci. Rep. 10 (1). doi: 10.1038/s41598-020-67165-5 PMC7347919 32647192

[B8] Amdur M. J. Mullin K. R. Waters M. J. Puggioni D. Wojnar M. K. Gu M.. Chemical control of spin-lattice relaxation to discover a room temperature molecular qubit. Chem Sci. (2022) 13 (23), 7034–7045. doi: 10.1039/d1sc06130e PMC9200133 35774181

[B9] Bazalova O. Kvicalova M. Valkova T. Slaby P. Bartos P. Netusil R.. (2016). Cryptochrome 2 mediates directional magnetoreception in cockroaches. Proc. Natl. Acad. Sci. U.S.A. 113, 1660–1665. doi: 10.1073/PNAS.1518622113/SUPPL_FILE/PNAS.1518622113.SAPP.PDF 26811445 PMC4760799

[B10] Berman P. R. (2016). Introductory quantum mechanics: a traditional approach emphasizing connections with classical physics.

[B11] Binhi V. N. (2016). Primary physical mechanism of the biological effects of weak magnetic fields. Biophys. (Russian. Federation). 61, 170–176. doi: 10.1134/S000635091601005X

[B12] Binhi V. N. (2019). Nonspecific magnetic biological effects: A model assuming the spin-orbit coupling. J. Chem. Phys. 151. doi: 10.1063/1.5127972 31779321

[B13] Brautigam C. A. Smith B. S. Ma Z. Palnitkar M. Tomchick D. R. Machius M.. (2004). Structure of the photolyase-like domain of cryptochrome 1 from Arabidopsis thaliana. Proc. Natl. Acad. Sci. U.S.A. 101, 12142–12147. doi: 10.1073/PNAS.0404851101/ASSET/FC950B81-C1CA-4DFF-8A3C-85F105B2E949/ASSETS/GRAPHIC/ZPQ0330457400005.JPEG 15299148 PMC514401

[B14] Buchachenko A. (2016). Why magnetic and electromagnetic effects in biology are irreproducible and contradictory? Bioelectromagnetics 37, 1–13. doi: 10.1002/BEM.21947 26769167

[B15] Castelvecchi D. Gibney E. (2022). “Spooky” quantum-entanglement experiments win physics Nobel. Nature 610, 241–242. doi: 10.1038/D41586-022-03088-7 36195711

[B16] Chaves I. Pokorny R. Byrdin M. Hoang N. Ritz T. Brettel K.. (2011). The cryptochromes: blue light photoreceptors in plants and animals. Annu. Rev. Plant Biol. 62, 335–364. doi: 10.1146/ANNUREV-ARPLANT-042110-103759 21526969

[B17] Davies E. (2023). The decrease in diurnal oxygen production in elodea under the influence of high geomagnetic variability: the role of light, temperature and atmospheric pressure. Int. J. Biometeorol. 67 (5), 821–834. doi: 10.1007/s00484-023-02457-9 36973472 PMC10167113

[B18] Deviers J. Cailliez F. de la Lande A. Kattnig D. R. (2022). Anisotropic magnetic field effects in the re-oxidation of cryptochrome in the presence of scavenger radicals. J. Chem. Phys. 156, 25101. doi: 10.1063/5.0078115/2840120 35032990

[B19] Dhiman S. K. Galland P. (2018). Effects of weak static magnetic fields on the gene expression of seedlings of Arabidopsis thaliana. J. Plant Physiol. 231, 9–18. doi: 10.1016/J.JPLPH.2018.08.016 30199755

[B20] Dhiman S. K. Wu F. Galland P. (2023). Effects of weak static magnetic fields on the development of seedlings of Arabidopsis thaliana. Protoplasma 260. doi: 10.1007/S00709-022-01811-9 36129584

[B21] Dodson C. A. Hore P. J. Wallace M. I. (2013). A radical sense of direction: signalling and mechanism in cryptochrome magnetoreception. Trends Biochem. Sci. 38, 435–446. doi: 10.1016/J.TIBS.2013.07.002 23938034

[B22] Engels S. Schneider N. L. Lefeldt N. Hein C. M. Zapka M. Michalik A.. (2014). Anthropogenic electromagnetic noise disrupts magnetic compass orientation in a migratory bird. Nature 509, 353–356. doi: 10.1038/nature13290 24805233

[B23] Erdmann W. Idzikowski B. Kowalski W. Szymański B. Kosicki J. Kaczmarek Ł. (2017). Can the tardigrade hypsibius dujardini survive in the absence of the geomagnetic field? PloS One 12 (9), e0183380. doi: 10.1371/journal.pone.0183380 28886031 PMC5590818

[B24] Fedele G. Edwards M. D. Bhutani S. Hares J. M. Murbach M. Green E. W.. (2014a). Genetic analysis of circadian responses to low frequency electromagnetic fields in drosophila melanogaster. PloS Genet. 10, e1004804. doi: 10.1371/JOURNAL.PGEN.1004804 25473952 PMC4256086

[B25] Fedele G. Green E. W. Rosato E. Kyriacou C. P. (2014b). An electromagnetic field disrupts negative geotaxis in Drosophila via a CRY-dependent pathway. Nat. Commun. 5, 1–6. doi: 10.1038/ncomms5391 PMC4104433 25019586

[B26] Foley L. E. Gegear R. J. Reppert S. M. (2011). Human cryptochrome exhibits light-dependent magnetosensitivity. Nat. Commun. 2, 1–3. doi: 10.1038/ncomms1364 PMC3128388 21694704

[B27] Fromm J. Lautner S. (2006). Electrical signals and their physiological significance in plants. Plant Cell Environ. 30 (3), 249–257. doi: 10.1111/j.1365-3040.2006.01614.x 17263772

[B28] Galland P. Pazur A. (2005). Magnetoreception in plants. J. Plant Res. 118, 371–389. doi: 10.1007/S10265-005-0246-Y 16283069

[B29] Gegear R. J. Casselman A. Waddell S. Reppert S. M. (2008). Cryptochrome mediates light-dependent magnetosensitivity in Drosophila. Nature 454, 1014–1018. doi: 10.1038/NATURE07183 18641630 PMC2559964

[B30] Grüning G. Wong S. Y. Gerhards L. Schuhmann F. Kattnig D. R. Hore P. J. Solovyov I. A. Effects of dynamical degrees of freedom on magnetic compass sensitivity: a comparison of plant and avian cryptochromes. J Am Chem Soc (2022) 144 (50), 22902–22914. doi: 10.1021/jacs.2c06233 36459632

[B31] Hammad M. Albaqami M. Pooam M. Kernevez E. Witczak J. Ritz T.. (2020). Cryptochrome mediated magnetic sensitivity in: Arabidopsis occurs independently of light-induced electron transfer to the flavin. Photochem. Photobiol. Sci. 19, 341–352. doi: 10.1039/c9pp00469f 32065192

[B32] Herbel V. Orth C. Wenzel R. Ahmad M. Bittl R. Batschauer A. (2013). Lifetimes of arabidopsis cryptochrome signaling statesin vivo. The Plant Journal. 74 (4), 583–592. doi: 10.1111/tpj.12144 23398192

[B33] Hiscock H. G. Worster S. Kattnig D. R. Steers C. Jin Y. Manolopoulos D. E.. (2016). The quantum needle of the avian magnetic compass. Proc. Natl. Acad. Sci. 113 (17), 4634–4639. doi: 10.1073/pnas.1600341113 27044102 PMC4855607

[B34] Hong G. Pachter R. (2015). Photoactivation of cryptochromes from drosophila melanogaster and sylvia borin: Insight into the chemical compass mechanism by computational investigation. J. Phys. Chem. B. 119, 3883–3892. doi: 10.1021/JP508871H/ASSET/IMAGES/LARGE/JP-2014-08871H_0004.JPEG 25710635

[B35] Hong G. Pachter R. (2023). Effects of inter-radical interactions and scavenging radicals on magnetosensitivity: spin dynamics simulations of proposed radical pairs. Eur. Biophys. J. 52 (1-2), 27–37. doi: 10.1007/s00249-023-01630-7 36792823

[B36] Hore P. J. Mouritsen H. (2016). The radical-pair mechanism of magnetoreception. Annu. Rev. Biophys. 45, 299–344. doi: 10.1146/ANNUREV-BIOPHYS-032116-094545 27216936

[B37] Karki N. Vergish S. Zoltowski B. D. Brian Zoltowski C. D. Professor A. (2021). Cryptochromes: Photochemical and structural insight into magnetoreception. Protein Sci. 30, 1521–1534. doi: 10.1002/PRO.4124 33993574 PMC8284579

[B38] Karogodina T. Y. Dranov I. G. Sergeeva S. V. Stass D. V. Steiner U. E. (2011). Kinetic magnetic-field effect involving the small biologically relevant inorganic radicals NO and O2.–. ChemPhysChem 12, 1714–1728. doi: 10.1002/CPHC.201100178 21598373

[B39] Karogodina T. Y. Sergeeva S. V. Stass D. V. (2009). Magnetic field effect in the reaction of recombination of nitric oxide and superoxide anion. Appl. Magn. Reson. 36, 195–208. doi: 10.1007/S00723-009-0018-2/FIGURES/3

[B40] Kattnig D. R. Hore P. J. (2017). The sensitivity of a radical pair compass magnetoreceptor can be significantly amplified by radical scavengers. Sci. Rep. 7, 1–12. doi: 10.1038/s41598-017-09914-7 28912470 PMC5599710

[B41] Kirschvink J. L. Gould J. L. (1981). Biogenic magnetite as a basis for magnetic field detection in animals. Biosystems 13, 181–201. doi: 10.1016/0303-2647(81)90060-5 7213948

[B42] Kominis I. K. (2009). Quantum Zeno effect explains magnetic-sensitive radical-ion-pair reactions. Phys. Rev. E. Stat. Nonlin. Soft. Matter. Phys. 80, 56115. doi: 10.1103/PHYSREVE.80.056115/FIGURES/4/MEDIUM 20365051

[B43] Lambert N. Chen Y. N. Cheng Y. C. Li C. M. Chen G. Y. Nori F. (2012). Quantum biology. Nat. Phys. 9, 10–18. doi: 10.1038/nphys2474

[B44] Lee A. A. Lau J. C. Hogben H. J. Biskup T. Kattnig D. R. Hore P. J.. (2014). Alternative radical pairs for cryptochrome-based magnetoreception. J R Soc Interface. 11 (95), 20131063. doi: 10.1098/rsif.2013.1063 24671932 PMC4006233

[B45] Liu Q. Wang Q. Deng W. Wang X. Piao M. Cai D.. (2017). Molecular basis for blue light-dependent phosphorylation of Arabidopsis cryptochrome 2. Nat. Commun. 8. doi: 10.1038/NCOMMS15234 PMC5437284 28492234

[B46] Lüdemann G. Solov’yov I. A. Kubař T. Elstner M. (2015). Solvent driving force ensures fast formation of a persistent and well-separated radical pair in plant cryptochrome. J. Am. Chem. Soc. 137, 1147–1156. doi: 10.1021/JA510550G/SUPPL_FILE/JA510550G_SI_004.PDF 25535848

[B47] Ma X. Zhang Q. Zhu Q. Liu W. Chen Y. Qiu R.. (2015). A robust CRISPR/cas9 system for convenient, high-efficiency multiplex genome editing in monocot and dicot plants. Mol. Plant 8, 1274–1284. doi: 10.1016/J.MOLP.2015.04.007 25917172

[B48] Maeda K. Henbest K. B. Cintolesi F. Kuprov I. Rodgers C. T. Liddell P. A.. (2008). Chemical compass model of avian magnetoreception. Nature 453, 387–390. doi: 10.1038/nature06834 18449197

[B49] Maeda K. Robinson A. J. Henbest K. B. Hogben H. J. Biskup T. Ahmad M.. (2012). Magnetically sensitive light-induced reactions in cryptochrome are consistent with its proposed role as a magnetoreceptor. Proc. Natl. Acad. Sci. U.S.A. 109, 4774–4779. doi: 10.1073/PNAS.1118959109/-/DCSUPPLEMENTAL/APPENDIX.PDF 22421133 PMC3323948

[B50] Maffei M. E. (2014). Magnetic field effects on plant growth, development, and evolution. Front. Plant Sci. 5. doi: 10.3389/FPLS.2014.00445/BIBTEX PMC4154392 25237317

[B51] Mani T. (2022). Molecular qubits based on photogenerated spin-correlated radical pairs for quantum sensing. Chem. Phys. Rev. 3 (2). doi: 10.1063/5.0084072

[B52] McFadden J. Al-Khalili J. (2016). Life on the edge: the coming of age of quantum biology.

[B53] Mouritsen H. Heyers D. Güntürkün O. (2016). The neural basis of long-distance navigation in birds. Annu. Rev. Physiol. 78, 133–154. doi: 10.1146/ANNUREV-PHYSIOL-021115-105054 26527184

[B54] Mouritsen H. Hore P. J. (2012). The magnetic retina: light-dependent and trigeminal magnetoreception in migratory birds. Curr. Opin. Neurobiol. 22, 343–352. doi: 10.1016/J.CONB.2012.01.005 22465538

[B55] Müller P. Ahmad M. (2011). Light-activated cryptochrome reacts with molecular oxygen to form a flavin-superoxide radical pair consistent with magnetoreception. J. Biol. Chem. 286, 21033–21040. doi: 10.1074/JBC.M111.228940 21467031 PMC3122164

[B56] Müller P. Bouly J. P. Hitomi K. Balland V. Getzoff E. D. Ritz T.. (2014). ATP binding turns plant cryptochrome into an efficient natural photoswitch. Sci. Rep. 4 (1), 5175. doi: 10.1038/srep05175 24898692 PMC4046262

[B57] Müller P. Yamamoto J. Martin R. Iwai S. Brettel K. (2015). Discovery and functional analysis of a 4th electron-transferring tryptophan conserved exclusively in animal cryptochromes and (6-4) photolyases. Chem. Commun. 51, 15502–15505. doi: 10.1039/C5CC06276D 26355419

[B58] Nicolaides C. Bazzi F. Vouros E. Flesariu D. F. Chrysochos N. Koutentis P. A.. (2023). Metal-free organic radical spin source. Nano. Lett. 23 (10), 4579–4586. doi: 10.1021/acs.nanolett.3c01044 37154760 PMC10214490

[B59] Nießner C. Denzau S. Peichl L. Wiltschko W. Wiltschko R. (2014). Magnetoreception in birds: I. Immunohistochemical studies concerning the cryptochrome cycle. J. Exp. Biol. 217, 4221–4224. doi: 10.1242/JEB.110965 25472972 PMC4254396

[B60] Nießner C. Denzau S. Stapput K. Ahmad M. Peichl L. Wiltschko W.. (2013). Magnetoreception: activated cryptochrome 1a concurs with magnetic orientation in birds. J. R. Soc. Interface 10. doi: 10.1098/RSIF.2013.0638 PMC3785833 23966619

[B61] Nohr D. Franz S. Rodriguez R. Paulus B. Essen L. O. Weber S.. (2016). Extended electron-transfer in animal cryptochromes mediated by a tetrad of aromatic amino acids. Biophys. J. 111, 301. doi: 10.1016/J.BPJ.2016.06.009 27463133 PMC4968396

[B62] Ozturk N. (2017). Phylogenetic and functional classification of the photolyase/cryptochrome family. Photochem. Photobiol. 93, 104–111. doi: 10.1111/PHP.12676 27864885

[B63] Parmagnani A. S. D’Alessandro S. Maffei M. E. (2022). Iron-sulfur complex assembly: Potential players of magnetic induction in plants. Plant Sci. 325, 111483. doi: 10.1016/J.PLANTSCI.2022.111483 36183809

[B64] Pinzon-Rodriguez A. Muheim R. (2017). Zebra finches have a light-dependent magnetic compass similar to migratory birds. J. Exp. Biol. 220, 1202–1209. doi: 10.1242/jeb.148098 28356366

[B65] Player T. C. Hore P. J. (2019). Viability of superoxide-containing radical pairs as magnetoreceptors. J. Chem. Phys. 151. doi: 10.1063/1.5129608/198329 31837685

[B66] Pooam M. Arthaut L.-D. Burdick D. Link J. Martino C. F. Ahmad M. (2019). Magnetic sensitivity mediated by the Arabidopsis blue-light receptor cryptochrome occurs during flavin reoxidation in the dark. Planta 249, 319–332. doi: 10.1007/s00425-018-3002-y 30194534

[B67] Pooam M. El-Esawi M. Aguida B. Ahmad M. (2020). Arabidopsis cryptochrome and Quantum Biology: new insights for plant science and crop improvement. J. Plant Biochem. Biotechnol. 29, 636–651. doi: 10.1007/s13562-020-00620-6

[B68] Procopio M. Link J. Engle D. Witczak J. Ritz T. Ahmad M. (2016). Kinetic modeling of the arabidopsis cryptochrome photocycle: FADH° accumulation correlates with biological activity. Front. Plant Sci. 7. doi: 10.3389/FPLS.2016.00888/BIBTEX PMC4924484 27446119

[B69] Qin S. Yin H. Yang C. Dou Y. Liu Z. Zhang P.. (2016). A magnetic protein biocompass. Nat. Mater. 15, 217–226. doi: 10.1038/NMAT4484 26569474

[B70] Ritz T. Adem S. Schulten K. (2000). A model for photoreceptor-based magnetoreception in birds. Biophys. J. 78, 707–718. doi: 10.1016/S0006-3495(00)76629-X 10653784 PMC1300674

[B71] Ritz T. Ahmad M. Mouritsen H. Wiltschko R. Wiltschko W. (2010). Photoreceptor-based magnetoreception: optimal design of receptor molecules, cells, and neuronal processing. J. R. Soc. Interface 7, S135. doi: 10.1098/RSIF.2009.0456.FOCUS 20129953 PMC2843994

[B72] Ritz T. Wiltschko R. Hore P. J. Rodgers C. T. Stapput K. Thalau P.. (2009). Magnetic compass of birds is based on a molecule with optimal directional sensitivity. Biophys. J. 96, 3451–3457. doi: 10.1016/J.BPJ.2008.11.072 19383488 PMC2718301

[B73] Schlosshauer M. (2007). Decoherence and the quantum-to-classical transition (Heidelberg: Springer Berlin). doi: 10.1007/978-3-540-35775-9_6

[B74] Schulten K. Swenberg C. E. Weiler A. (1978). A biomagnetic sensory mechanism based on magnetic field modulated coherent electron spin motion. Z. fur. Physikalische. Chemie. 111, 1–5. doi: 10.1524/ZPCH.1978.111.1.001/MACHINEREADABLECITATION/RIS

[B75] Sheppard D. Li J. Henbest K. Neil S. Maeda K. Storey J.. (2017). Millitesla magnetic field effects on the photocycle of an animal cryptochrome. Sci. Rep. 7, 1–7. doi: 10.1038/srep42228 28176875 PMC5296725

[B76] Silva J. Dobránszki J. (2015). Magnetic fields: how is plant growth and development impacted? Protoplasma 253 (2), 231–248. doi: 10.1007/s00709-015-0820-7 25952081

[B77] Solov’yov I. A. Chandler D. E. Schulten K. (2007). Magnetic field effects in arabidopsis thaliana cryptochrome-1. Biophys. J. 92, 2711–2726. doi: 10.1529/BIOPHYSJ.106.097139 17259272 PMC1831705

[B78] Solov’yov I. A. Schulten K. (2012). Reaction kinetics and mechanism of magnetic field effects in cryptochrome. J. Phys. Chem. B. 116, 1089–1099. doi: 10.1021/JP209508Y 22171949 PMC3266978

[B79] Wiltschko R. Ahmad M. Nießner C. Gehring D. Wiltschko W. (2016). Light-dependent magnetoreception in birds: the crucial step occurs in the dark. J. R. Soc. Interface 13. doi: 10.1098/RSIF.2015.1010 PMC4892254 27146685

[B80] Wiltschko W. Munro U. Ford H. Wiltschko R. (1993). Red light disrupts magnetic orientation of migratory birds. Nature 364, 525–527. doi: 10.1038/364525a0

[B81] Wiltschko R. Nießner C. Wiltschko W. (2021). The magnetic compass of birds: the role of cryptochrome. Front. Physiol. 12, 667000. doi: 10.3389/fphys.2021.667000 34093230 PMC8171495

[B82] Wiltschko W. Wiltschko R. (1981). Disorientation of inexperienced young pigeons after transportation in total darkness. Nature 291, 433–435. doi: 10.1038/291433a0

[B83] Wiltschko W. Wiltschko R. (1995). Migratory orientation of European robins is affected by the wavelength of light as well as by a magnetic pulse. J. Comp. Physiol. A. 177, 363–369. doi: 10.1007/BF00192425

[B84] Wiltschko W. Wiltschko R. (1999). The effect of yellow and blue light on magnetic compass orientation in European Robins, *Erithacus rubecula* . J. Comp. Physiol. A. 184, 295–299. doi: 10.1007/s003590050327

[B85] Wiltschko R. Wiltschko W. (2019). Magnetoreception in birds. J. R. Soc. Interface 16. doi: 10.1098/RSIF.2019.0295 PMC6769297 31480921

[B86] Worster S. Kattnig D. R. Hore P. J. (2016). Spin relaxation of radicals in cryptochrome and its role in avian magnetoreception. J. Chem. Phys. 145. doi: 10.1063/1.4958624 27448908

[B87] Xu C. Lv Y. Chen C. Zhang Y. Wei S. (2014). Blue light-dependent phosphorylations of cryptochromes are affected by magnetic fields in Arabidopsis. Adv. Space. Res. 53, 1118–1124. doi: 10.1016/J.ASR.2014.01.033

[B88] Xu J. Ping Y. (2023). Substrate effects on spin relaxation in two-dimensional Dirac materials with strong spin-orbit coupling. NPJ Comput. Mater. 9 (1), 47. doi: 10.1038/s41524-023-00992-y

[B89] Xu C. Yin X. Lv Y. Wu C. Zhang Y. Song T. (2012). A near-null magnetic field affects cryptochrome-related hypocotyl growth and flowering in Arabidopsis. Adv. Space. Res. 49, 834–840. doi: 10.1016/J.ASR.2011.12.004

[B90] Xu C. Yu Y. Zhang Y. Li Y. Wei S. (2016). Gibberellins are involved in effect of near-null magnetic field on arabidopsis flowering. Bioelectromagnetics 38 (1), 1–10. doi: 10.1002/bem.22004 27598690

[B91] Xu C. Yu Y. Zhang Y. Li Y. Wei S. (2017). Gibberellins are involved in effect of near-null magnetic field on Arabidopsis flowering. Bioelectromagnetics 38, 1–10. doi: 10.1002/BEM.22004 27598690

[B92] Xu C. Zhang Y. Yu Y. Li Y. Wei S. (2018). Suppression of Arabidopsis flowering by near-null magnetic field is mediated by auxin. Bioelectromagnetics 39, 15–24. doi: 10.1002/BEM.22086 28940601

[B93] Yamashita M. Tomita-Yokotani K. Hashimoto H. Takai M. Tsushima M. Nakamura T. (2004). Experimental concept for examination of biological effects of magnetic field concealed by gravity. Adv. Space. Res. 34, 1575–1578. doi: 10.1016/J.ASR.2004.01.022 15880894

